# A single-cell atlas and Shiny-based framework for murine lung injury and remodeling

**DOI:** 10.3389/fbinf.2026.1886929

**Published:** 2026-09-21

**Authors:** Qiuming Wang, Jixian Li, Philip J. Moos, Alessandro Venosa

**Affiliations:** 1 Department of Molecular Biosciences, University of California Davis School of Veterinary Medicine, Davis, CA, United States; 2 Texas Advanced Computing Center, The University of Texas at Austin, Austin, TX, United States; 3 Department of Pharmacology and Toxicology, University of Utah College of Pharmacy, Salt Lake City, UT, United States

**Keywords:** batch correction, cell-cell communication analysis, cell-type annotation, integration evaluation, lung injury and remodeling, R Shiny application, reference dataset, single-cell RNA sequencing (sc-RNA seq)

## Abstract

Single-cell RNA sequencing has provided unprecedented insights into the cellular heterogeneity of acute and chronic pulmonary injury. Lack of data-driven analytical frameworks remains a significant barrier to reproducible and biologically robust analyses. To address this challenge, we developed a modular, interactive analysis platform for single-cell RNA sequencing data processing and visualization. As a case study, we applied the framework to an immune-enriched murine lung dataset containing samples from different batches and across four conditions: control, inflammation and remodeling, fibrosis, and fibrotic lung with ozone exposure. Manual annotations performed on individual samples were used as ground-truth labels to evaluate automated cell-type annotations generated using the Tabula Muris Senis and LungMAP reference atlases. These annotations were also utilized to benchmark the performance of data preprocessing and integration combinations on batch-effect removal and the preservation of biological identity. Within the LIANA framework, we evaluated eight inference algorithms and 18 references for cell-cell communication analysis. In our results, we outlined the framework of the analysis pipeline and interactive platform. Through manual annotation, we identified 36 distinct cell populations, highlighting the limitations of automated reference-based annotations in resolving cell states missing from underlying references - particularly injury-induced monocyte-derived macrophages and eosinophils. In our dataset, Log-normalization outperformed SCTransform in both preserving cell-type separation and batch mixing, while FastMNN provided the best balance between technical batch correction and the retention of cell subtype identities. Cell-cell communication analysis revealed that permutation-based algorithms used by CellChat and CellPhoneDB improved prediction confidence, while MouseConsensus and OmniPath resources increased interaction coverage. These findings demonstrate that preprocessing, integration, and cell annotation strategies should be selected in a context-dependent, data-driven manner rather than relying on default workflows. The annotated murine lung dataset expands the existing atlas, and the modular interactive platform offers a practical framework to improve accessibility, reproducibility, and analytical precision in pulmonary research.

## 1 Introduction

Single-cell RNA sequencing (scRNA-seq) has emerged as an essential tool for characterizing cell type diversity and elucidating disease-associated mechanisms in acute and chronic pulmonary injury (Reyfman et al., 2019; Aran et al., 2019; Adams et al., 2020; Habermann et al., 2020; Wang et al., 2024). Over the past decade, rapid advancements in scRNA-seq technologies have been paralleled by the development of a wide array of computational methods for dimensionality reduction, clustering, cell-type annotation, integration, trajectory analysis, and inference of cellular communication (Dest et al., 2024; Arya et al., 2025; Molla Desta and Birhanu, 2025). Nonetheless, there is a paucity of comprehensive standardized pipelines that optimize the efficiency of these tools (Hou et al., 2020; Cheng et al., 2023).

A notable example pertains to data integration during the merging of multiple samples. Canonical Correlation Analysis (CCA), Reciprocal Principal Component Analysis (RPCA), Harmony, FastMNN (Fast Mutual Nearest Neighbors), and scVI (single-cell Variational Inference) are common methods designed to correct technical batch effects while preserving true biological variation (Butler et al., 2018; Hao et al., 2021; Korsunsky et al., 2019; Haghverdi et al., 2018; Lopez et al., 2018). However, the performance of these methods is not universally robust across all conditions (Cheng et al., 2023; Luecken et al., 2022; Tran et al., 2020). Use of different instruments, varying sequencing depths, and heterogeneity in sample dissociation and preparation across laboratories introduce complex technical noise (Luecken et al., 2022; Luecken and Theis, 2019; Tung et al., 2017). While dimension reduction techniques like Uniform Manifold Approximation and Projection (UMAP) are effective at visualizing large-scale batch effects (e.g., those arising from distinct sequencing depths), they remain qualitative diagnostics (Luecken et al., 2022; Luecken and Theis, 2019). Consequently, quantifying integration performance through objective metrics is essential to optimize data integration by minimizing technical variance without sacrificing the biological signal. With mild technical noise, determining whether integration is needed and which method to use is challenging because the analysis requires a delicate balance between alignment and preservation.

Cell-type annotation remains the most time-consuming step prior to downstream analyses, as current solutions heavily rely on reference datasets or established marker gene sets (Luecken and Theis, 2019; Abdelaal et al., 2019; Clarke et al., 2021). Although public data availability has improved significantly, high-quality reference datasets for disease-associated mouse lung tissue remain scarce. While the widely recognized Tabula Muris Senis (TMS) and LungMAP CellRefs (LungMAP) comprehensively cover the transcriptional landscape of the healthy lung across the lifespan, neither offers insights of lung injury and disease (Tabula Muris Consortium, 2020; Guo et al., 2023). This is significant because inflammatory disease is characterized by the influx of phenotypically active monocytes, which contribute directly to the inflammatory milieu and can replace the resident macrophage pool, alongside polymorphonuclear cells and T and B lymphocytes (Aran et al., 2019; Moos P. J. et al., 2024; Cembellin-Prieto et al., 2025). Prioritization of a specific cell type (e.g., alveolar or airway epithelial, fibroblast or myofibroblasts, interstitial or alveolar macrophages) influences the methodology of tissue dissociation (e.g. Dispase vs. Collagenase D, inclusion of DNase, use Fluorescence-Activated Cell Sorting) which are critical for cell viability and cell phenotype (Wang et al., 2024; Rodriguez et al., 2023; Rodríguez et al., 2025; Quintanal-Villalonga et al., 2022; Fujino et al., 2024). Thus, the choice of reference dataset and the underlying technical preparation may introduce significant bias, potentially compromising the accuracy of cell-type prediction and annotation across various methods.

Cell-cell communication (CCC) inferences provide a forecast of intercellular interactions in a technique that is inherently unable to capture post-transcriptional events. It involves leveraging computational models to integrate gene and gene-complex expression levels with curated ligand-receptor (LR) interaction resources. Heterogeneous curation of LR pairings across CCC resources (<15–20% overlap) and the paucity of species, tissue, or disease-specificity have been shown as constraints of its predictive power (Armingol et al., 2021; Jin et al., 2021; Efremova et al., 2020; Dimitrov et al., 2022). Evidence also highlights that the performance of these CCC inference tools is highly dependent on the computational method utilized, which produces conflicting “top-ranked” integrations when applied to the same dataset (Dimitrov et al., 2022). Thus, in the absence of systematic evaluation against biological ground truth or validated signaling networks, reliance on a single computational method or a resource may introduce significant bias, leading to the omission or misidentification of biological information.

Commercial platforms often provide user-friendly interfaces but frequently restrict the available methods for data normalization, integration, and CCC inference, or support direct comparisons between different algorithms (Best single; Wei et al., 2024). Among the six most popular scRNA-seq analysis platforms, only “Trailmaker” and “Partek Flow” allow selection of multiple integration algorithms, but neither platform quantifies the batch correction performance or the preservation of biological signal (Best single). Similarly, the incorporation of CCC inference tools by “Partek Flow” and “BBrowserX Pro” still lacks options for alternative resources or computational methods (CellPhoneDB; BBrowserX Pro). The code-based analysis pipelines offer greater flexibility but demand advanced programming and computational expertise, which is often a barrier for wet-bench researchers. Even for experienced programmers, manually repeating scripts during data exploration can introduce errors. Taken together, these limitations highlight the pressing need for an open-source platform that ensures a reproducible workflow, facilitates interactive data exploration, enables sharing with non-coding collaborators, and remains fully customizable for specific research requirements.

To address these critical gaps, our study introduces a high-resolution, annotated scRNA-seq dataset of a murine model of lung injury (Nureki et al., 2018). This resource extends beyond existing reference atlases by specifically incorporating multiple stages of inflammation and tissue remodeling. Utilizing this dataset as a representative case study, we demonstrate and explore key analytical steps in a data-driven scRNA-seq workflow, including cell-type annotation, normalization and transformation, data integration, and CCC inferences. In addition, providing the research community with a reusable and flexible platform, we developed an interactive R Shiny application. This platform enables researchers to explore complex lung fibrosis scRNA-seq data and perform validated analyses regardless of their programming and computational expertise. This comprehensive resource is strategically positioned to support future studies in lung biology, systems immunology, and computational method development.

## 2 Results

### 2.1 Single cell RNA sequencing analysis pipeline development

We established a standardized scRNA-seq analysis pipeline consisting of five sequential stages: (1) Quality Control (QC) and Filtering, (2) Cell-Type Annotation, (3) Data Integration, (4) Integration Evaluation, and (5) Downstream Analysis and Visualization (Figure 1A).

**FIGURE 1:**
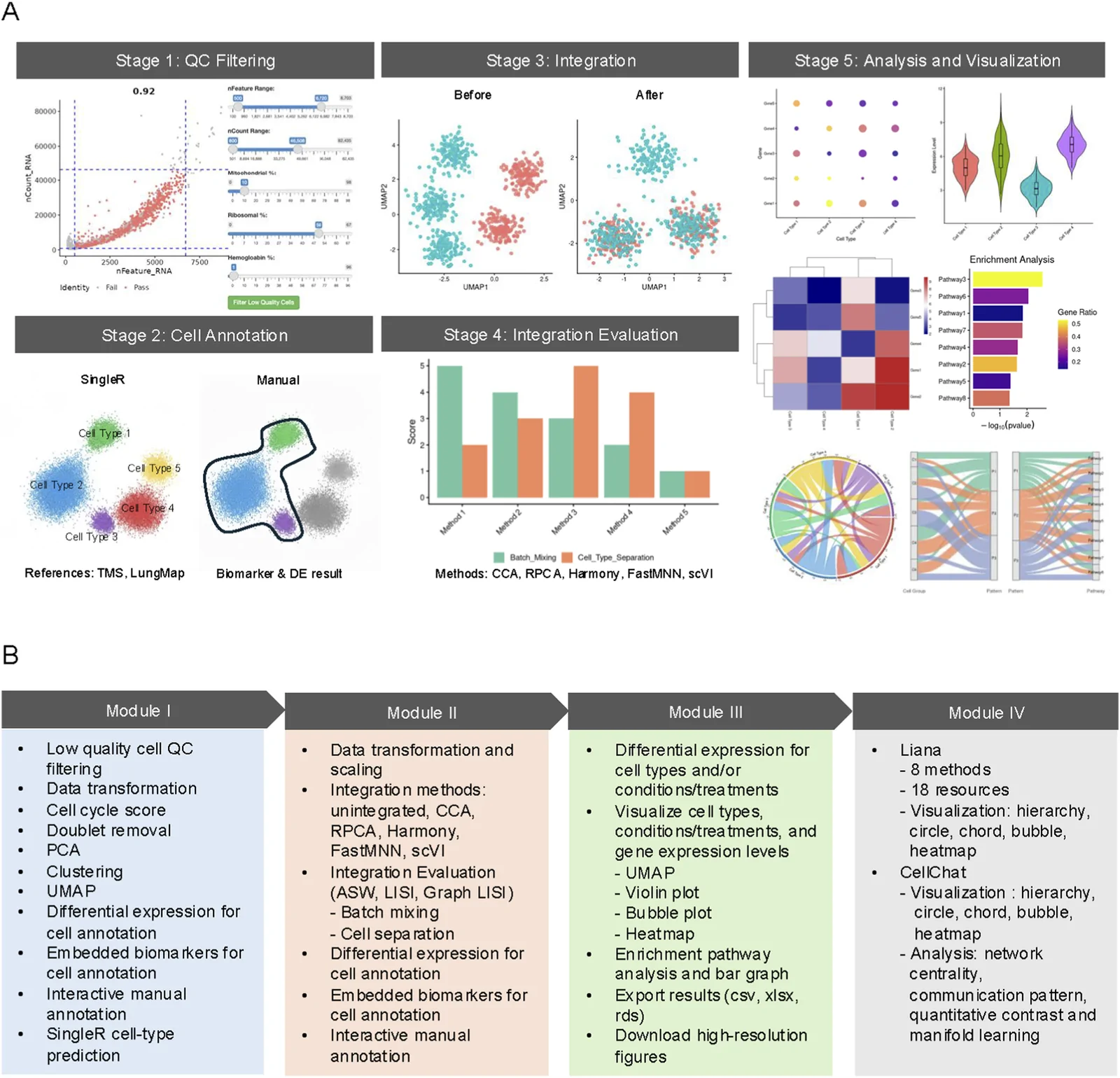
Standardized scRNA-seq analysis pipeline and interactive user interface. **(A)** Schematic of the single-cell transcriptomic workflow. Five-stage analysis includes QC Filtering, Cell Annotation, Integration, Integration Evaluation, Analysis and Visualization. **(B)** Modules of the integrated Shiny application.

To process individual samples, the initial step applies stringent filters to exclude low-quality cells based on the number of detected features (*nFeature*), the total number of transcripts (*nCount*), and the percentage of reads mapping to mitochondrial, ribosomal, and hemoglobin genes (*Mitochondrial%, Ribosomal%, Hemoglobin%*). Doublet identification and removal are performed using the *DoubletFinder* algorithm (McGinnis et al., 2019). Next, cell types are annotated through either an automated or manual process. SingleR, an automated annotation tool embedded, assigns identities by calculating the highest correlation between individual single-cell transcriptomes and reference datasets. The manual process requires a combination of data processing methods, including PCA, clustering, and UMAP; cells are labeled using known biomarkers (Aran et al., 2019).

Once annotated, processed data from individual samples are merged and batch-corrected through the Data Integration and Integration Evaluation stages of the pipeline. The final Downstream Analysis and Visualization stage of the pipeline is designed to address core biological questions through differential expression (DE) analysis, pathway enrichment analysis, CCC inferences, supported by visualization techniques for both gene expression levels and high-level analytical results.

### 2.2 Interactive analysis platform development

To standardize this pipeline and ensure it is accessible, reproducible, and time-efficient, we developed an interactive R Shiny application. The application is organized into four functional modules: (I) Individual sample QC, Filtration, and Cell Annotation, (II) Data Integration and Evaluation, (III) Data Analysis and Visualization, and (IV) Cell-Cell Communication (Figure 1B). The first module performs initial QC filtering and doublet removal, followed by data preprocessing, PCA, clustering, and UMAP generation. An interactive UMAP interface allows users to zoom, pan, overlay biomarker expression levels, or SingleR prediction results. It further enables manual selection of cell subsets for refined reanalysis and subtype annotation (e.g., clustering, UMAP generation, DE analysis).

Processed data from individual samples are merged in the second module, where the user can choose from multiple integration methods based on the specific requirements for batch correction. To guide the selection of the optimal method, the platform provides an interactive UMAP display, overlaying batch identity and integrated coordinates, alongside quantified evaluation scores for batch-mixing and cell-type separation. The third module facilitates deep data exploration and figure generation. It offers a range of analytical tools and customization options for producing high-resolution figures suitable for reports, grants, and publications. The final module is dedicated to CCC inferences. The App features both CellChat v2, the primary visualization and analysis tool, alongside the LIANA framework, which incorporates eight computational methods and 18 interaction resources (Dimitrov et al., 2022; Jin et al., 2025).

### 2.3 Sftpc^I73T^ lung reference dataset construction and characterization

We characterized a comprehensive reference dataset using a previously established murine model of inflammatory fibrogenic exacerbations, driven by a clinically relevant mutation in the Surfactant Protein C gene (*Sftpc* ^*I73T*^) (Nureki et al., 2018; Cheminant et al., 2025). The study investigated four distinct conditions: steady state (Control), inflammation and early remodeling occurring 2 weeks post injury (Inflam), established fibrosis at 6 weeks (Fib), and fibrosis with ozone exposure (Fib_O3) (Supplementary Table S1). Our analysis annotated 36 distinct cellular identities encompassing both unique cell types and distinct activation states of common populations (Figures 2A,B; Table 1; Supplementary Table S2). Cell identities were confirmed by evaluating top unique gene signatures for each population (Figure 2C). Cellular profiling revealed 22 annotated subtypes of immune cells alongside a sparse recovery of alveolar and ciliated epithelial cells, likely stemming from the mild digestive profile of Collagenase D (Table 1).

**FIGURE 2:**
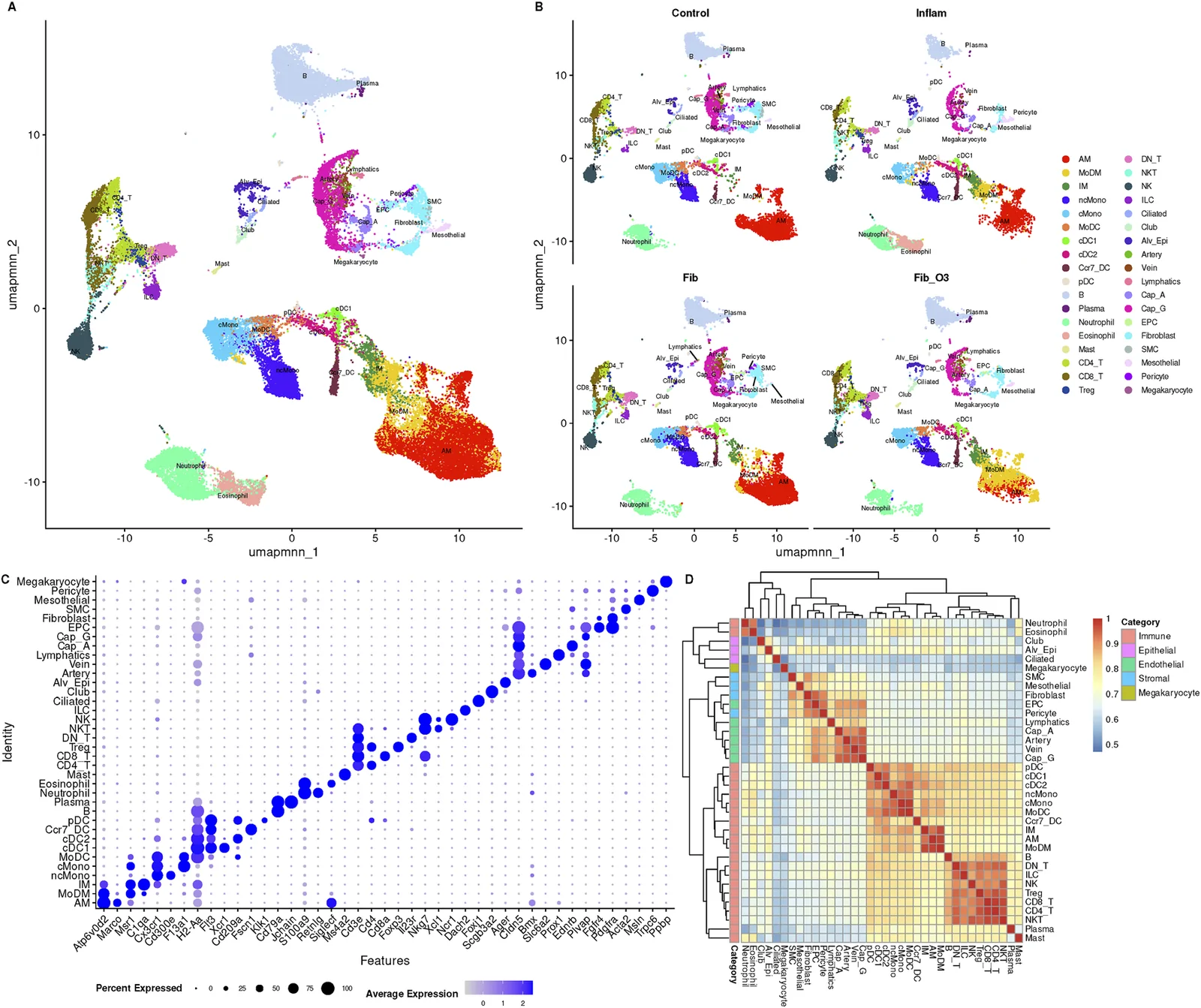
Integrated single-cell transcriptomic landscape of the SftpcI73T murine lung model. **(A)** Uniform Manifold Approximation and Projection (UMAP) of 45,197 cells integrated via FastMNN, color-coded by identified cell type. **(B)** Split UMAP projections visualizing cell density and distribution across four experimental conditions: Steady-state (Control), Tamoxifen-induced inflammation and remodeling (Inflam), fibrosis (Fib), and ozone exposure in fibrotic lung (Fib_O3). **(C)** Bubble plot displaying canonical marker gene expression (x-axis) across cell types (y-axis). Dot size represents the percentage of cells expressing the marker, and color intensity indicates the mean expression level. **(D)** Hierarchical clustering of cell type transcriptional correlations using Euclidean distance. The left annotation bar indicates five broad lineage categories. Color scale (red-to-blue) represents Pearson correlation coefficients.

**TABLE 1:** Distribution of cell types across the scRNA-seq dataset.

Cell category	Full nomenclature	Label	Count	Freq (%)
Immune	Alveolar macrophage	AM	7,588	16.79
Immune	Monocyte-derived macrophage	MoDM	2,997	6.63
Immune	Interstitial macrophage	IM	1,060	2.35
Immune	Classical monocyte	cMono	2,519	5.57
Immune	Non-classical monocyte	ncMono	2,997	6.63
Immune	Ccr7 dendritic cell	Ccr7_DC	535	1.18
Immune	Classical dendritic cell subset 1	cDC1	473	1.05
Immune	Classical dendritic cell subset 2	cDC2	652	1.44
Immune	Monocyte-derived dendritic cell	MoDC	472	1.04
Immune	Plasmacytoid dendritic cell	pDC	95	0.21
Immune	Natural killer cell	NK	1825	4.04
Immune	Natural killer T cell	NKT	500	1.11
Immune	Regulatory T cell	Treg	305	0.67
Immune	CD4^+^ T cell	CD4_T	2095	4.64
Immune	CD8^+^ T cell	CD8_T	1985	4.39
Immune	Double_negative T cell	DN_T	745	1.65
Immune	Innate lymphoid cell	ILC	515	1.14
Immune	Eosinophil	Eosinophil	924	2.04
Immune	Neutrophil	Neutrophil	3,968	8.78
Immune	B cell	B	6,109	13.52
Immune	Plasma cell	Plasma	52	0.12
Immune	Mast cell	Mast	148	0.33
Epithelial	Alveolar epithelial cell	Alv_Epi	542	1.2
Epithelial	Club cell	Club	352	0.78
Epithelial	Ciliated cell	Ciliated	135	0.3
Endothelial	Lymphatic endothelial cell	Lymphatics	150	0.33
Endothelial	Arterial endothelial cell	Artery	124	0.27
Endothelial	Venous endothelial cell	Vein	281	0.62
Endothelial	General capillary cell	Cap_G	2,908	6.43
Endothelial	Aerocyte capillary cell	Cap_A	495	1.1
Endothelial	Endothelial progenitor cell	EPC	40	0.09
Stromal	Fibroblast	Fibroblast	1,240	2.74
Stromal	Smooth muscle cell	SMC	53	0.12
Stromal	Mesothelial cell	Mesothelial	174	0.38
Stromal	Pericyte	Pericyte	115	0.25
Megakaryocyte	Megakaryocyte	Megakaryocyte	29	0.06

Summary of annotated cell populations in the SP-C^I73T^ dataset. Table includes cell type category, full nomenclature, and corresponding labels used for downstream analysis. The count and relative frequency of the cells are provided for each cell population.

Mapping of Alveolar Macrophages (AM), Monocyte-Derived Macrophages (MoDM), and Interstitial Macrophages (IM) into a FastMNN-integrated UMAP space revealed evident population shifts across conditions (Figure 2B). As expected, MoDMs were largely absent in steady-state Control but exhibited a progressive increase through the Inflam and Fib stages, reaching peak abundance in the Fib_O3 group (Figure 2B). Moreover, the UMAP embedding highlighted the transient appearance of blood-derived eosinophils in the Inflam study group, underscoring their specific recruitment during the early remodeling phase (Moos P. J. et al., 2024).

Bi-clustered heatmap showed strong positive transcriptional similarity among myeloid (macrophages, monocytes, and dendritic cells) and lymphoid populations (B, T, natural killer cells, innate lymphoid cells) (Figure 2D). In contrast, plasma cells segregated independently of the broader B cell cluster. This separation reflected their specialized secretory transcriptome dedicated to antibody production (Luecken and Theis, 2019). Similarly, neutrophils and eosinophils formed a distinct cluster sequestered from other immune populations. This independent grouping is attributed to the unique granulocyte gene signature and low RNA content inherent to these cell types (Figure 2D) (Zilion et al., 2019). Among the non-immune compartments, Endothelial Progenitor Cells (EPCs) clustered more closely with stromal populations than with mature Endothelial compartments (Figure 2D). This likely reflects a shared mesenchymal-like transcriptional program characteristic of fibrosis (Tsukui et al., 2020). Finally, megakaryocytes and ciliated epithelial cells showed generally relatively low correlations with other populations, though ciliated cells maintained moderate correlation with alveolar epithelial (Alv_Epi) and club cells (Figure 2D).

### 2.4 Impact of references atlas composition on automated annotation performance

To assess the utility of existing community resources for automated annotation of injured lung dataset, we utilized SingleR to label our *Sftpc* ^*I73T*^ dataset using two established references: TMS and LungMAP. We then used our manual annotations as the ground truth to evaluate the predictive performance of these automated annotations. SingleR identified 29 cell types using TMS and 37 using LungMAP. The matched nomenclature across these annotations is summarized in Supplementary Table S3. While a common set of 17 major cell types was shared across the manual and automated annotations, only B cells, NK cells, and lymphatics endothelial cells demonstrated high predictive sensitivity and precision across both references. Injury-specific populations arising from blood populations (MoDM and eosinophils) were uniquely identified through our manual annotation. The majority of MoDMs were misclassified as either AMs or IMs by both references; monocyte-derived dendritic cells (MoDCs) were labeled as monocytes by TMS and as Type 2 Conventional Dendritic Cells (cDC2) by LungMap. This inaccurate annotation led to high sensitivity but low precision of AM predictions and low sensitivity and precision of IM prediction in both references. Both references also misidentified eosinophils for neutrophils, likely due to shared granulocytic signatures that are not sufficiently distinguished in steady state atlases (Figures 2A, 3A,B).

**FIGURE 3:**
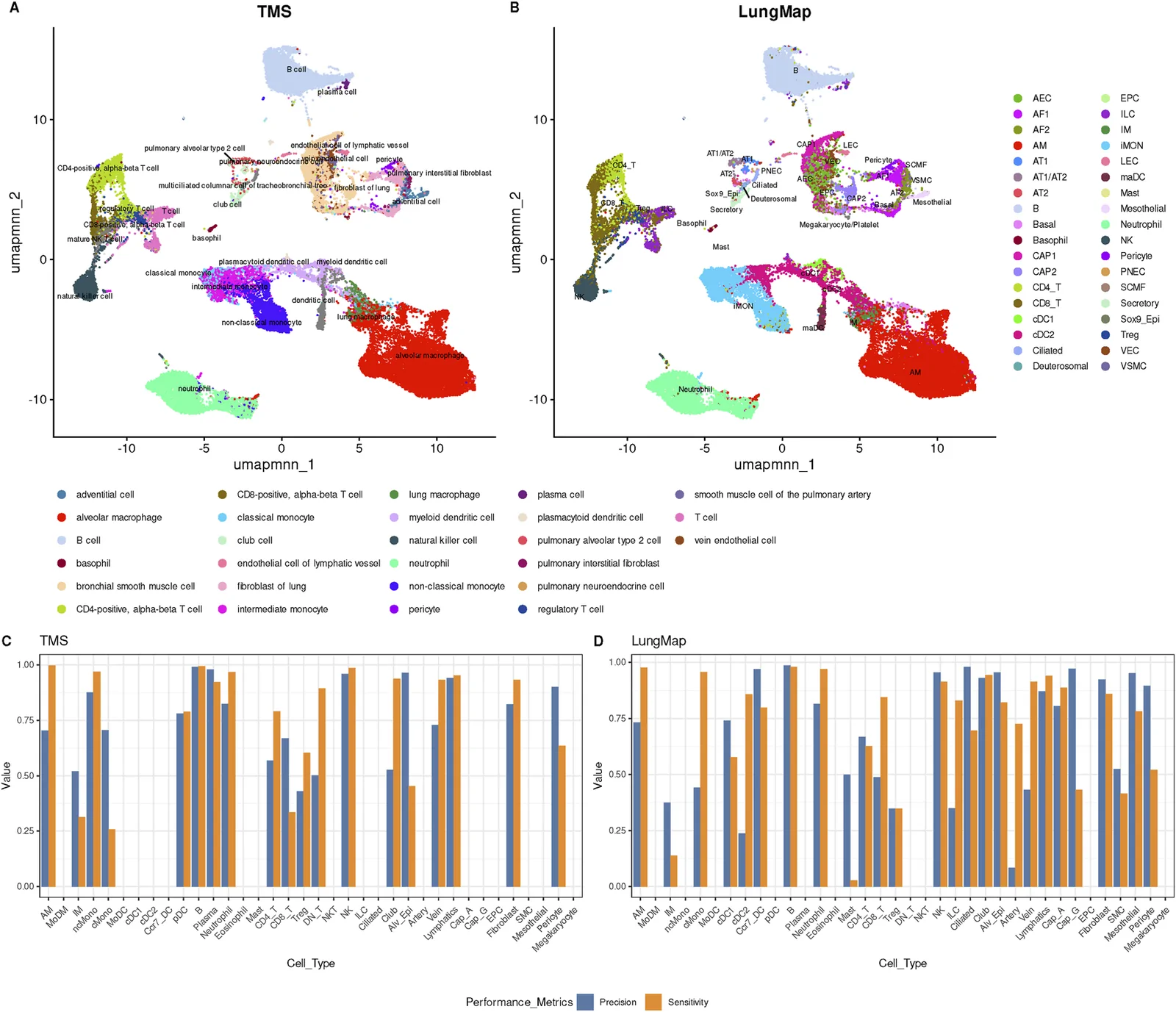
SingleR automated annotation of lung injury and remodeling dataset using Tabula Muris Senis and LungMAP steady-state reference atlases. **(A)** UMAP visualization of the *Sftpc* ^*I73T*^ dataset with cell identities predicted by SingleR using the Tabula Muris Senis (TMS) steady-state reference. Cell types are indicated by the color legend provided below the plot. **(B)** UMAP visualization with identities predicted using the LungMAP reference. The color legend is provided to the right of the plot. In both **(A,B)**, colors are harmonized with manual annotations for common lineages to highlight discrepancies in cell-type prediction. **(C,D)** Quantitative assessment of prediction precision (blue) and sensitivity (yellow) for TMS **(C)** and LungMAP **(D)** references against manual ground-truth labels. Missing bars indicate cell populations in the lung injury and fibrosis dataset that lacked a corresponding or sufficiently matched cell type in the respective reference dataset.

The TMS reference exhibited significant limitations across immune and endothelial lineages (Figure 3C; Supplementary Table S4). Among monocyte subtypes, TMS frequently misclassified the manually annotated classical monocytes (cMono) as “intermediate,” which led to low sensitivity of the cMono prediction, but non-classical monocytes (ncMono) were accurately predicted (Figures 2A, 3A,C). Additionally, TMS failed to distinguish between Type 1 Conventional Dendritic Cells (cDC1) and cDC2 subtypes and missed multiple immune populations, such as ILCs and Mast cells (Figures 2A, 3A,C). Predictive performance was also poor for Artery, Capillary General (Cap_G), and Alveolar Capillary (Cap_A) endothelial cells, which were mislabeled as “smooth muscle cells of the pulmonary artery” and “bronchial smooth muscle cells” (Figures 2A, 3A,C). Finally, TMS annotated Mesothelial and Endothelial Progenitor Cells (EPC) as fibroblasts due to the absence of these cell types in it, which reduced predictive precision of the fibroblast category (Figures 2A, 3A,C).

The LungMAP reference demonstrated stronger predictive power among epithelial, stromal, and endothelial compartments but exhibited deficient performance in predicting immune cell types (Figure 3D; Supplementary Table S5). Beyond its inability to resolve MoDMs and eosinophils, LungMap failed to identify MoDCs, ncMono, Plasmacytoid Dendritic Cells (pDC), plasma cells, Double-Negative T cells (DN_T), and NKT cells (Figure 3D). Lastly, LungMAP mislabeled Cap_G cells as Vein, Artery, and EPC, while two subtypes of Smooth Muscle Cells (SMC) and Pericytes were erroneously annotated as Fibroblasts (Figures 2A, 3B).

### 2.5 Benchmarking data preprocessing and integration workflows

To mitigate technical variation across the five collection batches, we conducted a systematic benchmarking of ten integration workflows pair with two data transformation and five integration methods (Supplementary Table S1). In UMAP embeddings, only the SCTransformed unintegrated data exhibited prominent batch-related color shifts (Figures 4A–F; Supplementary Figure S1). Our quantitative evaluation of batch-effect removal (batch-mixing) and preservation of biological identity (cell-type separation) were based on Average Silhouette Width (ASW), Local Inverse Simpson’s Index (LISI), and Graph Local Inverse Simpson’s Index (GLISI) quantitative matrices. Experimental batch information generated during library preparation and sequencing was used as the batch labels for batch-mixing evaluation, whereas manually annotated cell-type labels established prior to data transformation and integration were used to calculate the cell-type separation score as an independent measure of biological signal preservation. The median was applied to represent each matrix (Table 2). Under the unintegrated circumstances, Log-normalization provided a better batch mixing and cell-type separation rank scores compared to SCTransform across most of the quantitative matrices (Figures 4G,H; Table 2). When applying the same integration method across both transformation methods, log-normalization consistently yielded higher performance rank scores, suggesting it provided a more stable foundation for our dataset (Figures 4G,H; Table 2).

**FIGURE 4:**
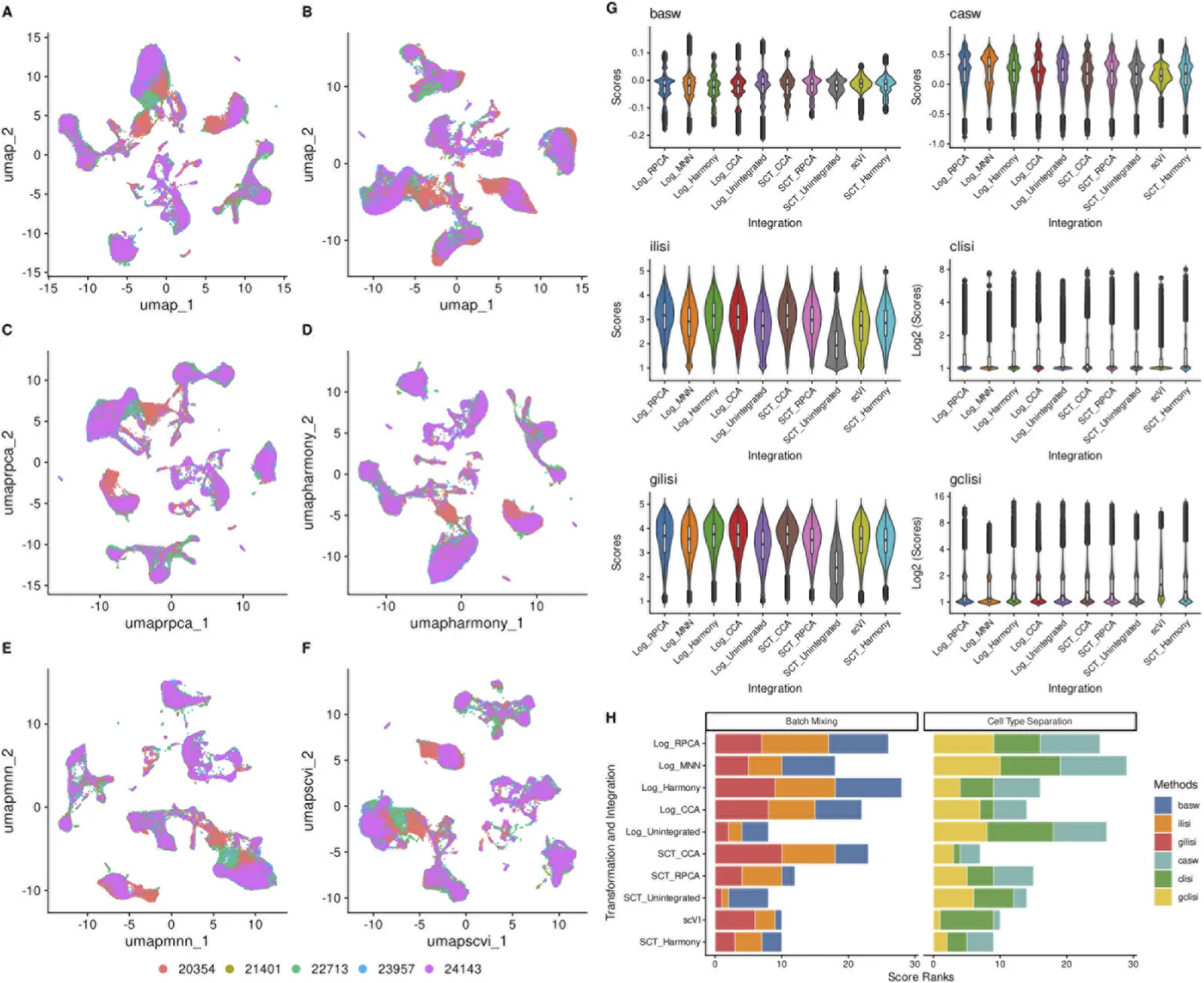
Benchmarking of data transformation and integration workflows for the *Sftpc* ^*I73T*^ dataset. (**A–F)** UMAP visualizations of six representative workflows demonstrating batch distribution (colored by n = 5 batches). Workflows include: **(A)** log-normalization (unintegrated), **(B)** SCTransform (unintegrated), **(C)** log-normalization with RPCA, **(D)** log-normalization with Harmony, **(E)** log-normalization with fastMNN, and **(F)** scVI (raw counts input). **(G)** Quantitative performance metrics across 10 evaluated workflows. Metrics include Average Silhouette Width (ASW), Local Inverse Simpson’s Index (LISI), and Graph Local Inverse Simpson’s Index (Graph LISI) for both batch mixing (basw, ilisi, gilisi) and cell-type separation (casw, clisi, gclisi). Due to highly skewed distributions, clisi and gclisi scores are displayed on a log2 scale. **(H)** Integrated ranking of workflows based on median scores across all metrics. Bars are colored by quantitative performance metrics and separated by metric category (batch mixing vs. cell-type separation). Workflows were ordered from top to bottom in the bar graph by the total sum of rank scores, identifying the performance from the best to the worst.

**TABLE 2:** Comparative performance ranking of scRNA-seq integration workflows.

Integration	basw	ilisi	gilisi	casw	cilisi	gcilisi
Log_unintegrated	4	2	2	8	10	8
Log_cca	7	7	8	5	2	7
Log_rpca	9	10	7	9	7	9
Log_harmony	10	9	9	7	5	4
Log_mnn	8	5	5	10	9	10
SCT_unintegrated	6	1	6	2	6	1
SCT_cca	5	8	1	3	1	6
SCT_rpca	2	6	10	6	4	3
SCT_harmony	3	4	4	4	3	5
scVI	1	3	3	1	8	2

Median-based rank scores are provided for combinations of data transformation methods (Log-normalization, SCTransform) and integration algorithms (e.g., harmony, FastMNN, etc.). Note that scVI, was evaluated using raw counts as input. Performance is scored on a scale of 1–10, where a score of 1 represents the poorest performance and 10 represents the highest integration efficacy.

Among the methods tested, RPCA achieved the highest aggregated rank scores, offering the most balanced between batch-mixing and cell-type separation quantitatively (Figures 4G,H; Table 2). When analyzed individually, Harmony demonstrated the most aggressive batch correction, whereas FastMNN proved to be the best for preserving distinct cell-type separation (Figures 4G,H; Table 2). FastMNN was utilized to display our *Sftpc* ^*I73T*^ dataset, because it provided the clearest resolution of macrophage subtypes across different conditions, with minimal overlap in UMAP space (Figures 2A,B; Supplementary Figures S2, S3). Surprisingly, scVI yielded lower overall rank scores but preserved condition-specific macrophage subtypes in UMAP (Table 2; Supplementary Figure S4). It produced narrow distributions of Batch ASW (basw) and Cell ASW (casw) and significantly reduced the skewness of cell-type GLISI (gclici) compared to other methods (Figures 4G,H; Table 2). This suggests that scVI exerts strong global regularization on the latent space.

### 2.6 CCC inference methods and resources benchmarking via LIANA

Given that CCC inferences are influenced by LR database curations (resource) and computational models (method), we applied the LIANA framework to systematically evaluate their impacts on the *Sftpc* ^*I73T*^ dataset (Dimitrov et al., 2022). We evaluated five re-implemented methods (*connectome, logfc, natmi, sca,* and *cellphonedb*) and three external methods (*CytoTalk*, *CellChat*, and *iTalk)* as defined in the LIANA framework. The re-implemented methods yielded a consistent number of total interactions, and they were shared across methods (Figure 5A). Only *cellphonedb* accounted for multi-subunit complexes, while other re-implemented methods were post-aggregated (Dimitrov et al., 2022). Among the external methods, *CytoTalk* and *CellChat* incorporated multi-subunit complexes (Figure 5A). Notably, *iTalk* predicted an extremely large amount of total and unique interactions, likely due to its reliance on expression enrichment rather than stringent matching criteria (Figure 5A) (Dimitrov et al., 2022; Wang et al., 2019). *CellChat* and *cellphonedb* provide a higher confidence signaling landscape by performing permutation tests to mitigate false-positive predictions (Dimitrov et al., 2022).

**FIGURE 5:**
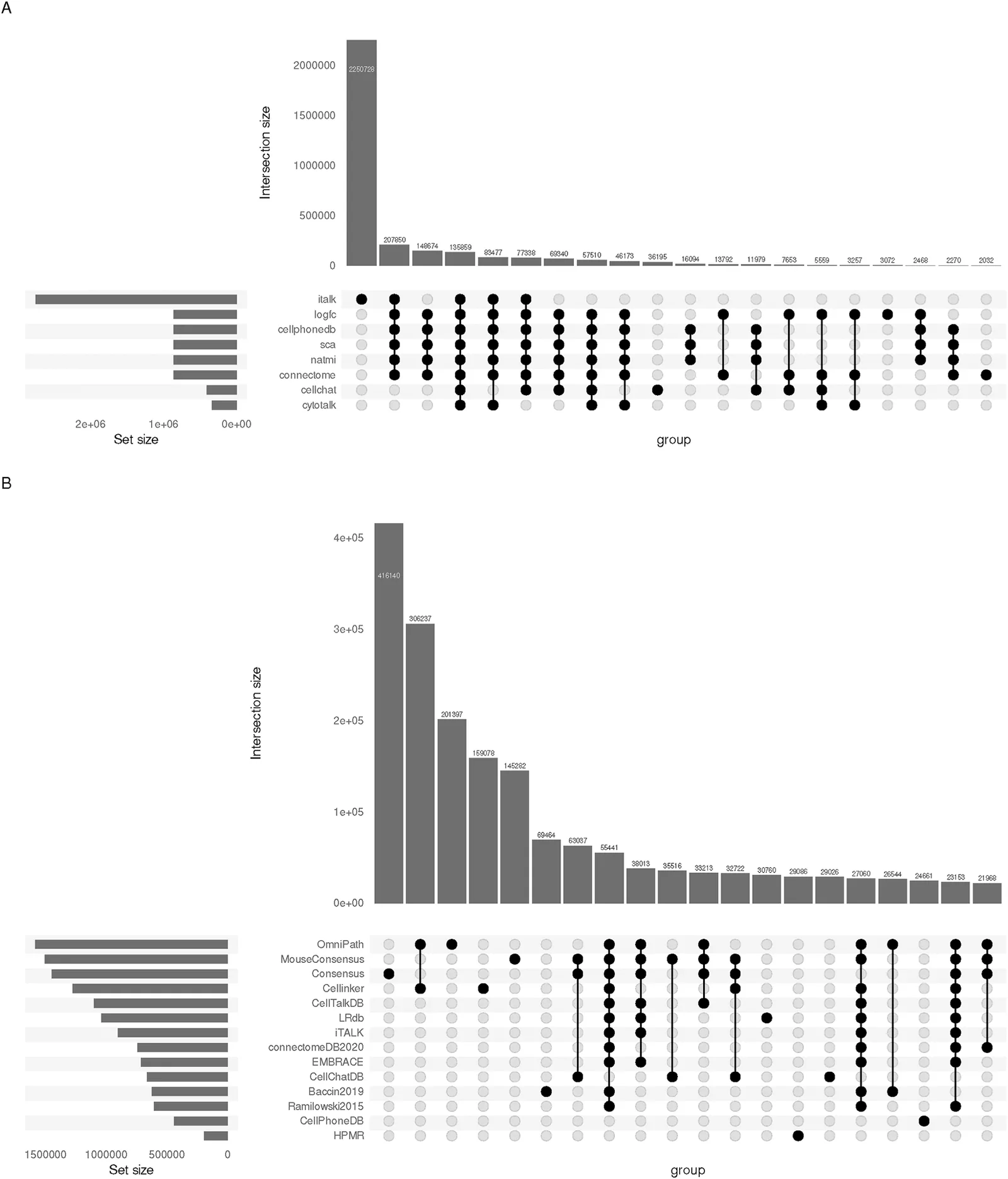
Comparative analysis of Cell-Cell Communication (CCC) inference resources and methods within the LIANA framework. **(A)** Upset plot displaying the overlap of unique ligand-receptor interactions across 14 resources. All 18 resources were integrated, but only 14 resources were showed due to limited intersection groups. Redundant interactions identified by multiple methods were collapsed to ensure unique entries. The top bar graph indicates the number of unique or shared interactions (intersection size) for the top 25 intersection groups. The bottom dot matrix identifies the specific combination of resources contributing to each intersection. The left horizontal bar graph represents the total number of interactions cataloged per resource (set size), ranked from highest to lowest. **(B)** Upset plot illustrating the overlap of predicted interactions across CCC inference methods. Redundant interactions across resources were removed to compare method-specific predictions. The top, bottom, and left plots follow the same logic as in **(A)**.

Among the 18 accessible resources through LIANA, MouseConsensus is the only natively murine; the remaining 17 are human-derived. To facilitate a broader comparison of these resources regardless of reference method, we mapped the *Sftpc* ^*I73T*^ dataset to human orthologs and converted back after the prediction generated, retaining only genes with one-to-one matches. OmniPath yielded the highest total number of interactions and second highest count of unique interactions. It shared a large core of interactions with Celllinker, MouseConsensus, and Consensus (Figure 5B). Consensus featured the largest group of unique interactions, nearly double that of the next highest resources, indicating a high degree of exclusive content (Figure 5B). CellLinker involved the greatest diversity of unique ligands and receptors within the *Sftpc* ^*I73T*^ dataset (Supplementary Figure S5, S6). MouseConsensus, Consensus, CellChatDB, CellPhoneDB, ICELLNET, Baccin 2019, and Cellinker provide complex-containing interactions (Supplementary Figure S5B, S6B). MouseConsensus demonstrated enrichment in total and unique interactions, ligands, receptors, and multi-subunit complexes, highlighting the biological value of species-specific curation (Figure 5B; Supplementary Figure S5, S6). Furthermore, while the Baccin2019 resource was smaller in size, it exhibited a high percentage of unique interactions, ligands, receptors, and multi-subunit complexes compared to larger consensus resources (Figure 5B; Supplementary Figure S5, S6).

## 3 Discussion

Standardized analytical pipelines for scRNA-seq data are essential for improving computational efficiency and ensuring reproducible discovery across datasets. However, existing frameworks often lack the flexibility to support data-driven method optimization tailored to specific biological contexts, such as interspecies and organ-level variability, or disease models. To address these challenges, our study was designed with two primary objectives: first, the generation of a high-resolution and immune-focused scRNA-seq atlas detailing the progression of murine pulmonary injury; and second, the development of an interactive R Shiny platform to democratize advanced computational approaches and facilitate systematic context-aware method evaluation.

Our analysis revealed that automated cell-type prediction using healthy murine lung references (TMS and LungMAP) was inadequate to identify multiple immune sub-populations and disease-related monocyte-derived macrophages and eosinophils. Our manually annotated *Sftpc* ^*I73T*^ dataset attempts to addresses this gap by gathering information from publish scRNA-seq datasets of murine injury and therefore standardize the identification of populations engaged in sterile injury and lung remodeling (Reyfman et al., 2019; Adams et al., 2020; Habermann et al., 2020; Joshi et al., 2020). By combining cells from the Bronchoalveolar Lavage (BAL) with collagenase D digested lung tissue, our protocol intentionally enriched the dataset for immune populations, while the inclusion of multiple injury states expanded the diversity of observed cellular subtypes. Utilizing our atlas as a reference for automated cell annotation tools enhances the prediction power for future immune-focused queries in respiratory research. Despite the depth of our immune profiling, the *Sftpc* ^*I73T*^ dataset has limitations regarding mesenchymal, epithelial, and endothelial cell diversity due to the specific tissue processing methodology employed. A recent series of studies by Rodríguez et al. utilized a similar Tamoxifen-induced *Sftpc* ^*I73T*^ model with a primary emphasis on mesenchymal and epithelial populations via Dispase digestion and CD45^+^ cell depletion, provides a complementary reference for cross-study integration (Rodriguez et al., 2023; Rodríguez et al., 2025). This dataset also contains the steady-state and 2-week post-tamoxifen timepoint, which can serve as biological anchor for batch integration.

To expand the lineage map of the injured lung, the strategic integration of external datasets is necessary. A similar conceptual framework has been published by Sikkema et al., which focused on the re-annotation of human samples from the Human Lung Cell Atlas (Sikkema et al., 2023). However, such an endeavor brings up challenges in optimizing dataset selection, mitigating batch effects, and maintaining computational efficiency as data volume increases. To maintain a compact and performant reference, we recommend downsampling the reference to include only samples specifically matched to the query dataset. Recent reports support the notion that restricting the reference to highly variable features (HVGs) effectively reduces the feature space without sacrificing resolution (Zhao et al., 2025). More broadly, the performance of reference-based cell annotation is fundamentally determined by the biological composition of the reference atlas, not solely by the prediction algorithm itself. Since no single reference represents all biological states, our pipeline combines automated multi-atlas annotation with manual refinement using established marker genes. This strategy leverages the strengths of diverse references while minimizing the limitations of any single atlas for heterogeneous or injury-associated cell populations.

Although SCTransform is often recommended for its ability to stabilize variance and account for varying sequencing depth, our results reveal a notable departure from its widespread adoption as the preferred preprocessing methodology (Hafemeister and Satija, 2019; Germain et al., 2020). In our experiments using the *Sftpc* ^*I73T*^ dataset, Log-normalization outperformed SCTransform in both batch mixing and cell-type separation. This discrepancy likely stems from their distinct mathematical foundations: whereas preserves the relative expression structure after Log-normalization, allowing downstream highly variable gene selection and PCA to capture biologically informative variation; SCTransform’s regularized Negative Binomial model scales the Pearson residuals to a unit variance of one across all features (Hafemeister and Satija, 2019; Townes et al., 2019). This global variance stabilization may increase the influence of sampling noise in lowly expressed genes (Luecken et al., 2022), thereby distorting the biological signal in PCA and UMAP space and making it harder for integration tools to distinguish batch effects from cell-type-specific biological signals (Ahlmann-Eltze and Huber, 2023). Furthermore, because high-performing methods like FastMNN were originally optimized log-normalized data, the choice of transformation fundamentally dictates the success of downstream integration (Haghverdi et al., 2018). These findings suggest that dataset-specific evaluation of normalization and transformation methods is necessary to preserve biological resolution in downstream analyses.

The manually curated cell-type labels were established on individually processed samples before dataset merging and integration workflows. Our comparison of five widely utilized integration methods revealed a clear trade-off between technical batch correction and biological preservation. The contracting performance of Harmony and FastMNN highlights the risks of excessive batch correction on resolution of subtle cell states and rare populations. We found that quantitative metrics such as LISI, Graph LISI, and ASW should be used in conjunction with visualization inspection tools (e.g. UMAP) for optimal integration method selection. Notably, although RPCA achieved the highest aggregate quantitative rank by balancing batch-mixing and lineage preservation, FastMNN ultimately provided the most biologically meaningful UMAP embedding. This discrepancy highlights a limitation: aggregate scoring systems often weigh each metric equally, which can lead to results biased by subtle numerical differences or specific experimental conditions. In our study, the “Inflam” study group was collected as a single batch. This created a unique challenge since condition-specific populations (e.g. eosinophils) inherently penalize “batch-mixing” scores, as there are no corresponding cells in other batches available for mixing. The scVI has been reported as one of the best integration methods for large-scale atlases and cross-species context (Luecken et al., 2022; Song et al., 2023). The poor performance in our study suggests strong global regularization of latent space, likely reflecting the influence of abundant cell populations, such as macrophages. Further, our relatively small dataset contained less complex batch effect compared to large-scale atlases, which may have led to overfitting of the scVI model. The result of our comparison suggests that manual, sample-specific annotation performed prior to data merging and integration, together with the use of cell-type labels, an independent reference, are essential for evaluating integration performance, ensuring that rare or condition-specific identities are not sacrificed for global batch-homogenization.

In this work, we also comprehensively investigated multiple CCC inference methods using the LIANA framework and found that *CellChat* and *cellphonedb* methods employ permutation testing to reduce false-positive interactions among eight tested methods. However, the LIANA framework does not automatically use the most recently updated methods and resources of CellPhoneDB and CellChat. The Python-based CellPhoneDB v5 maintains its high-precision subunit architecture and expands its coverage to ∼3,000 interactions, including non-protein molecules such as steroid hormones and metabolites (Troulé et al., 2025). Its new CellSign feature is particularly powerful for linking ligand-receptor pairs to downstream transcription factor (TF) activity (Troulé et al., 2025). The R-based CellChat v2 models the global flow of information using mass-action logic and cofactors, and most importantly its paired CellChatDB includes a dedicated murine-based resource (Jin et al., 2021; Jin et al., 2025). Owing to its native murine resources, extensive visualization capabilities, and integrated network and pathway analysis tools, CellChat v2 was embedded within our R Shiny platform. Despite these benefits, our results also show that CellChat v2 generated relatively low total interaction output among the methods tested because of the stricter statistical modeling and pathway level aggregation. Our group has previously published CellChat-based cell-cell interactions in the inflamed lung. While this work predicted strong complement signaling, our expression analysis also revealed no ligand-receptor pairings for C1qa, C1qb, C1qc, resulting in a distorted network prediction for interstitial macrophages (Moos P. J. et al., 2024). These findings provide practical guidance for the CCC component of our analysis pipeline. To reduce the risks of missing potentially significant interactions for murine lung studies, we recommend pairing *CellChat* and *cellphonedb* methods with expanded databases, such as OminPath, MouseConsensus, Consensus, Cellinker, and Baccion2019, via the LIANA framework.

## 4 Conclusion

In this study, we established a high-resolution single-cell atlas of the *Sftpc* ^*I73T*^ murine model, providing a critical reference for the complex immune landscape characteristic of acute and chronic pulmonary injury. Our systematic benchmarking demonstrates that data normalization, transformation, and integration should be selected in a data-driven, context-dependent manner to balance between batch effect removal and biological signal preservation. Furthermore, we showed that combining statistically rigorous tools (*CellChat*, *cellphonedb*) with the broader molecular coverage of LIANA provides a practical strategy for improving qualitative and quantitative CCC inference in murine datasets. To facilitate the broader application of these results, we developed an interactive R Shiny platform that democratizes access to our optimized analytical pipelines. Ultimately, this study provides both a biological roadmap and a data-driven analytical framework for respiratory single-cell research, enabling researchers to systematically evaluate and select appropriate computational methods for their own datasets while maintaining a reproducible workflow.

This R Shiny application provides an accessible implementation of the proposed analytical framework, enabling researchers with limited programming experience to systematically evaluate alternative analytical strategies. By enforcing a traceable workflow, this platform promotes reproducibility and advances data exploration. Our future work will focus on four key directions: 1) validation of the tool with other murine models of pulmonary disease; 2) incorporation of spatial transcriptomics datasets by implementing advanced tools like LIANA+ (Dimitrov et al., 2024); 3) refinement of the machine learning models for automated annotation and biomarker discovery; and 4) extrapolation and authentication of murine cellular annotation markers and signaling pathways in the human lung. In parallel with these efforts, expansion of this application to extra-pulmonary fibrosing systems (e.g. liver, kidney) may promote scientific collaboration and further accelerate discovery.

## 5 Materials and methods

### 5.1 Mouse model and sample collection

The samples utilized in these studies are obtained from surfactant protein (SP)-C mutant mice. Our group has previously developed and characterized the inflammatory and fibrogenic events occurring during the tamoxifen-dependent induction of the most common disease-linked mutation in the gene encoding for the SP-C (Nureki et al., 2018). Four experimental conditions (n = 2 for each) were utilized to validate our framework: air-exposed controls (Control); 2-week post-tamoxifen-induced SP-C mutant injury, consistent with the peak of inflammation and early lung remodeling (Inflam); 6-week post-tamoxifen-induced SP-C mutant injury, consistent with established fibrosis (Fib); 6-week post-tamoxifen induced fibrosis also challenged with the air pollutant ozone (Fib-O3). Following bronchoalveolar lavage collection, the perfused lung was collected and enzymatically processed (Collagenase D, 2 mg/mL), as previously described (Moos P. J. et al., 2024). Single cell suspensions were achieved through mechanical mincing of the tissue and passage through a 70 μm cell strainers. Single cell suspension and bronchoalveolar lavage were then combined and counted for the following sequencing steps.

### 5.2 Single cell RNA library preparation and sequencing

Samples were processed across five independent batches (Batch IDs: 24143, 20354, 21401, 22713, 23957) at the High-Throughput Genomics Shared Resource at the University of Utah (Huntsman Cancer Institute). Single-cell library preparation was performed using the 10x Genomics Chromium Single Cell 3′ GEM Library and Gel Bead Kit v3 to enable 3′ digital gene expression profiling. Suspensions containing approximately 800 to 16,000 cells per sample were loaded into a Chromium Chip B with partitioning oil, reverse transcription reagents, and gel beads containing 3.5 million unique 10x Barcodes. Gel Beads-in-emulsion (GEMs) were generated during a 9-min run on the 10x Genomics Chromium Controller, facilitating the co-encapsulation of single cells with barcoded beads. Within these droplets, poly-adenylated mRNA was reverse-transcribed, incorporating a unique 10x Cell Barcode and Unique Molecular Identifier (UMI) to each cDNA molecule for cell-specific transcript assignment. Following GEM breakage and library construction, library quality control was conducted, which included a Qubit dsDNA Assay, Agilent DNA ScreenTape Assay, and Kapa Library Quantification by qPCR to qualify and normalize the library pool. Final libraries were sequenced on an Illumina NovaSeq 6000 platform using a paired-end configuration. Sequencing was performed in blocks of at least 100 million read pairs to ensure sufficient depth for high-resolution transcriptomic analysis. Raw sequencing data (FASTQ files) were aligned to the GRCm38 (mm10) reference genome using the 10X Genomics Cell Ranger (v7.0) count pipeline.

### 5.3 Modular workflow and computational environment design

The interactive R Shiny platform was intentionally designed as a modular workflow to accommodate different computational environments while improving memory management and computational efficiency. Module I (individual sample quality control, filtering, and cell annotation) operates on individual samples and is designed to run on a personal computer. Module II (data integration and evaluation) and Module IV (cell-cell communication analysis) computing part contains the most computationally intensive analyses and is therefore recommended for Linux-based servers or high-performance computing (HPC) environments. To minimize computational requirements for downstream analyses, the outputs from Module II retain only the information required for subsequent analyses, enabling Module III (data analysis and visualization) and Module IV (cell-cell communication analysis) visualization part to run efficiently on personal computers. This modular design allows users to perform routine analyses locally while reserving server or HPC resources for computationally intensive integration tasks.

### 5.4 Data quality control (QC) and filtering

To ensure high data quality, cells were filtered based on the following criteria: mitochondrial gene expression <10%, ribosomal gene expression <50%, hemoglobin gene expression <1%, and unique feature counts >500 gene per cell. Potential doublets were identified and excluded using the DoubletFinder algorithm (McGinnis et al., 2019). To account for transcriptional heterogeneity driven by cell proliferation, were assigned cell cycle score using *CellCycleScoring* function in Seurat v5.0, utilizing the gene sets defined by (Hao et al., 2024; Tirosh et al., 2016).

### 5.5 Data transformation and scaling

To compare the impact of normalization on integration, two methods were utilized: Log-Normalization and SCTransform. Log-Normalization scales the raw counts to 10, 000 and natural-log transformed using “LogNormalize” option with *NormalizeData* function in Seurat (Butler et al., 2018). SCTransform applies a regularized negative binomial regression to stabilize variance and remove technical artifacts associated with sequencing depth (Chou et al., 2022; Hafemeister and Satija, 2019). For both methods, technical covariant (mitochondrial gene percentage and cell cycle score) were regressed to minimize their influence on following dimension reduction and clustering analysis.

### 5.6 Dimensionality reduction and clustering

Following normalization and regression of technical covariates, dimensionality reduction was performed using Principal Component Analysis (PCA) on the scaled expression matrix restricted to highly variable genes (HVGs). HVGs were identified within each dataset to enrich biologically informative features while minimizing stochastic noise from low-abundance transcripts. The top 30 principal components (PCs) were retained for downstream analyses based on variance structure and stability of clustering results across a range of dimensional cutoffs. PCA served to compress the high-dimensional transcriptomic space into a reduced representation that preserves dominant sources of biological variation while attenuating gene-level noise. This reduced space formed the basis for neighborhood graph construction and subsequent clustering. Cellular relationships were modeled using a Shared Nearest Neighbor (SNN) graph constructed in PCA space. By emphasizing shared local neighborhood structure, the SNN framework enhances robustness to technical noise and prevents artificial merging of transcriptionally distinct populations (Butler et al., 2018). Unsupervised clustering was performed in Seurat using the Leiden community detection algorithm applied to the SNN graph to identify groups of transcriptionally similar cells (Butler et al., 2018; Waltman and Van Eck, 2013). Clustering resolution was systematically tuned to balance lineage-level separation with detection of biologically meaningful subpopulations. For lineage-specific analyses, subsets were iteratively reprocessed (HVG identification, PCA, graph construction, clustering) to resolve finer cellular states. For visualization, Uniform Manifold Approximation and Projection (UMAP) was applied to the PCA space to generate two-dimensional embeddings. UMAP is a non-linear dimensionality reduction method that preserves local neighborhood relationships while maintaining the global structure of the data manifold, enabling intuitive visualization of cellular population structure in complex transcriptomic datasets (McInnes et al., 2020).

### 5.7 SingleR cell type prediction

We utilized SingleR to assign cell identities by calculating Spearman rank correlations between cells of our *Sftpc* ^*I73T*^ murine dataset and two established references: LungMap Mouse Lung CellRef (LungMap) and Tabula Muris Senis (TMS). LungMap is a comprehensive scRNA-seq reference build from 17 mouse lung samples that cover embryonic day (E) 16.5 to postnatal day (PND) 28 at eight developmental time points (E16 n = 2, E18 n = 2, PND1 n = 2, PND3 n = 2, PND7 n = 3, PND10 n = 2, PND14 n = 2, PND28 n = 2) (Guo et al., 2023). It encompassed two lineage levels (major cell type) and three refined cell type levels, up to 40 distinct mouse lung cell types (Guo et al., 2023). TMS is a large-resolution scRNA-seq atlas of aging in the mouse from 1 month to 30 months (1-month M n = 2, 3-month F n = 1, 3-month M n = 2, 18-month F n = 2, 18-month M n = 2, 21-month F n = 2, 30-month M n = 4) in 23 tissues, including the lung. It contains 30 different cell-type labels (Tabula Muris Consortium, 2020). In this study, we extracted droplet-based (10X Genomics) lung tissue data from the entire reference.

### 5.8 Manual cell type annotation

Annotation was refined through an iterative process within our R Shiny application. Initial major cell types were identified using canonical biomarkers. Subpopulations were further resolved by employing an interactive free-hand lasso tool (via Plotly) to select specific clusters for re-PCA, re-clustering, and re-UMAP until all subtypes were expertly identified and validated against differential expression results and biomarkers in the literature (Guo et al., 2023; Moos P. et al., 2024; Tabula Muris Consortium, 2018; Duong et al., 2022; Ghaedi et al., 2020; Zhang et al., 2022; Jirmo et al., 2023; Negretti et al., 2021).

### 5.9 Transcriptional similarity analysis

Transcriptional similarity among manually annotated cell populations was evaluated using aggregated expression profiles. Raw counts were summed across all cells within each cell population using Seurat’s AggregateExpression function and subsequently log-normalized. Pairwise similarity was calculated using Pearson correlation across genes. The resulting correlation matrix was visualized as a bi-clustered heatmap using Euclidean distance and complete-linkage hierarchical clustering. Aggregation across cells reduced the influence of stochastic cell-level dropout on the similarity estimates.

### 5.10 Benchmarking of integration methods

We evaluated five commonly used integration methods implemented in the Seurat v5 framework to correct for technical variation across five experimental batches and four disease conditions: Canonical Correlation Analysis (CCA), Reciprocal Principal Component Analysis (RPCA), Harmony, Fast Mutual Nearest Neighbors (FastMNN), and single-cell Variational Inference (scVI). These methods were selected as they represent widely applied integration strategies available within a unified analytical framework. CCA is an anchor-based method that aligns datasets by identifying shared correlation structures across features and projecting them into a common space (Butler et al., 2018).

RPCA extends this framework by identifying anchors through reciprocal projection between datasets, improving computational efficiency, and reducing the risk of overcorrection in large or heterogeneous datasets (Hao et al., 2021). Harmony performs batch correction through iterative adjustment of cell embeddings, enabling alignment across batches while preserving global and local data structure (Korsunsky et al., 2019). FastMNN corrects batch effects by identifying mutual nearest neighbors across datasets and aligning cells based on local similarity, which helps maintain separation of biologically distinct populations (Haghverdi et al., 2018). scVI is a deep generative modeling approach that learns a batch-corrected latent representation directly from raw count data (Lopez et al., 2018). By modeling gene expression using a probabilistic framework, it accounts for technical variability while capturing underlying biological structure (Lopez et al., 2018). The resulting latent space is used for downstream visualization and clustering.

### 5.11 Quantitative evaluation of data transformation and integration

The *Sftpc* ^*I73T*^ dataset was generated across five batches, with Inflam samples (n = 2) originating from a single batch and Control, Fib, and Fib_O3 samples (n = 2 each) distributed across separate batches (Supplementary Table S1). To systematically assess the impact of preprocessing and integration, we evaluated 10 workflows comprising two data transformation methods (Log-Normalization and SCTransform) and five integration methods (CCA, RPCA, Harmony, FastMNN, and scVI). Integration performance was assessed using both qualitative and quantitative criteria. UMAP visualization was used for initial inspection of batch mixing and cluster structure, while quantitative evaluation was performed using Average Silhouette Width (ASW), Local Inverse Simpson’s Index (LISI), and Graph LISI (GLISI). These metrics were selected to jointly evaluate two key objectives: (i) effective removal of technical variation (batch mixing) and (ii) preservation of biological identity (cell-type separation). Manual cell-type annotations and batch labels were used as ground truth for evaluation. LISI-based metrics quantified local neighborhood diversity, with batch LISI (iLISI) assessing the extent of mixing across batches and cell-type LISI (cLISI) evaluating the purity of cell-type neighborhoods (Luecken et al., 2022). Graph LISI extended this framework to the Shared Nearest Neighbor (SNN) graph, providing a topology-aware measure of integration quality that is less sensitive to distortions from low-dimensional embeddings (Luecken et al., 2022). ASW was used as a complementary metric to evaluate both cell-type separation and batch mixing (Luecken et al., 2022). It quantifies the relative distance of each cell to its assigned group compared to neighboring groups, providing a measure of cluster cohesion and separation. For cell-type labels, higher ASW values indicate well-separated and cohesive clusters, reflecting strong preservation of biological identity. In contrast, for batch labels, lower ASW values indicate effective mixing, as cells are not preferentially grouped by batch. Thus, while ASW captures the same two evaluation objectives as LISI, its interpretation depends on the label context and the directionality of the score. To enable comparison across metrics with different scales, we applied a median-based ranking approach, assigning relative scores (1–10) to each workflow for each metric. These rankings were then aggregated to identify the optimal combination of transformation and integration methods (Table 2).

### 5.12 Cell-cell communication (CCC) inference

To evaluate the coverage of intercellular communication inference, we employed the LIANA framework to conduct a systematic comparison across multiple computational methods and ligand-receptor (LR) resources. The methodological comparison included eight distinct algorithms: Connectome, logFC, NATMI, SingleCellSignalR (SCA), CellPhoneDB, CytoTalk, CellChat, and iTALK. Simultaneously, we assessed the content and overlap of 18 independent LR databases, including Cellinker, OmniPath, Consensus, MouseConsensus, CellTalkDB, LRdb, ConnectomeDB2020, Baccin2019, iTALK, Ramilowski2015, EMBRACE, CellChatDB, HPMR, ICELLNET, Guide2Pharma, and Kirouac2010. To compare inference methods independently of the underlying resources, each method was evaluated across all 18 LR resources, and duplicate interactions identified across resources were collapsed before summarization. Conversely, to compare LR resources independently of the inference methods, each resource was evaluated using all eight methods, and duplicate interactions identified across methods were collapsed. The analysis focused on quantifying the structural differences in LR pair composition, specifically evaluating the diversity of identified ligands, receptors, and their respective multimeric complexes across frameworks. Method comparisons were summarized by the total predicted interactions, whereas resource comparisons additionally evaluated the total and unique ligands, receptors, ligand subunits, and receptor subunits. The intersections of unique and shared interactions, ligands, receptors, ligand subunits, and receptor subunits were visualized using UpSet plots. To highlight the primary drivers of variability or consensus among the tools, visualization was restricted to the top 20 intersection groups.

### 5.13 Ortholog mapping and resource integration

For human-based resources, mouse genes were mapped to human orthologs. To leverage the comprehensive human-based ligand-receptor (LR) resources provided within the LIANA framework, we performed a systematic ortholog conversion of the murine gene expression data. We restricted our analysis to one-to-one orthologs between *Mus musculus* and *Homo sapiens* to ensure high confidence signaling predictions and avoid ambiguities associated with many-to-many gene relationships. Gene symbols were mapped using the internal mapping dictionaries provided by the OmniPath and LIANA R packages. During this conversion process, a total of 16,578 mouse genes were excluded due to a lack of a strict one-to-one human match. The resulting filtered expression matrix was then utilized as input for 17 human-centric resources. For the MouseConsensus resource, the analysis was performed using native murine gene symbols, identifying a core set of 96 CCC-related genes specifically involved in the mouse lung fibrosis context.

### 5.14 Software and environment

All analyses were conducted in R (v4.3.3) using RStudio (2025.5). The interactive platform was developed using Shiny. Key R packages included: Seurat (v5.3.1), SeuratObject (v5.3.0), SeuratWrapper (v0.3.0), SingleR (v2.10.0), DoubletFinder (v2.0.4), LIANA (v1.5.0), CellChat (v2.1.2), ggplot2 (v4.0.0), plotly (v4.10.4), glmGamPoi (1.18.0), DT (v0.35), tidyverse (v2.0.0).

## Data Availability

The raw sequencing data (FASTQ files) and the processed gene-cell expression matrices generated in this study have been deposited in the Gene Expression Omnibus (GEO) under accession number GSE247520 and GSE343116. The complete analytical pipeline used to generate the results in this study and the interactive R Shiny application, which supports the modular scRNA-seq workflow described in this work, is available on GitHub (https://github.com/qiumingwang925/scRNA-seq). Detailed documentation and a user guide are provided within the repository to ensure reproducibility and accessibility for researchers without extensive coding expertise.

## References

[B1] Abdelaal T. Michielsen L. Cats D. Hoogduin D. Mei H. Reinders M. J. T. (2019). A comparison of automatic cell identification methods for single-cell RNA sequencing data. Genome Biol. 20, 194. 10.1186/s13059-019-1795-z 31500660 PMC6734286

[B2] Adams T. S. Schupp J. C. Poli S. Ayaub E. A. Neumark N. Ahangari F. (2020). Single-cell RNA-seq reveals ectopic and aberrant lung-resident cell populations in idiopathic pulmonary fibrosis. Sci. Adv. 6, eaba1983. 10.1126/sciadv.aba1983 32832599 PMC7439502

[B3] Ahlmann-Eltze C. Huber W. (2023). Comparison of transformations for single-cell RNA-seq data. Nat. Methods 20, 665–672. 10.1038/s41592-023-01814-1 PMC10172138 37037999

[B4] Aran D. Looney A. P. Liu L. Wu E. Fong V. Hsu A. (2019). Reference-based analysis of lung single-cell sequencing reveals a transitional profibrotic macrophage. Nat. Immunol. 20, 163–172. 10.1038/s41590-018-0276-y 30643263 PMC6340744

[B5] Armingol E. Officer A. Harismendy O. Lewis N. E. (2021). Deciphering cell-cell interactions and communication from gene expression. Nat. Rev. Genet. 22, 71–88. 10.1038/s41576-020-00292-x 33168968 PMC7649713

[B6] Arya A. Tripathi P. Dubey N. Aier I. Kumar Varadwaj P. (2025). Navigating single-cell RNA-sequencing: protocols, tools, databases, and applications. Genomics Inf. 23, 13. 10.1186/s44342-025-00044-5 PMC12085826 40382658

[B7] BBrowserX pro: advanced analytical tool for single-cell RNA-sequencing data. BioTuring Documentation. Available online at: https://talk2data.bioturing.com/docs/?app=bbrowserxpro&document=01_bbxpro_intro (Accessed March 8, 2026).

[B8] Best single-cell RNA-sequencing data analysis tools in 2024. Available online at: https://www.biomage.net/blog/best-single-cell-rna-sequencing-data-analysis-tools-in-2024 (Accessed March 8, 2026).

[B9] Butler A. Hoffman P. Smibert P. Papalexi E. Satija R. (2018). Integrating single-cell transcriptomic data across different conditions, technologies, and species. Nat. Biotechnol. 36, 411–420. 10.1038/nbt.4096 29608179 PMC6700744

[B10] CellPhoneDB - Partek Flow User Manual. Available online at: https://help.partek.illumina.com/partek-flow/user-manual/task-menu/exploratory-analysis/cellphonedb (Accessed March 8, 2026).

[B11] Cembellin-Prieto A. Luo Z. Kulaga H. Baumgarth N. (2025). B cells modulate lung antiviral inflammatory responses via the neurotransmitter acetylcholine. Nat. Immunol. 26, 775–789. 10.1038/s41590-025-02124-8 PMC12043518 40263611

[B12] Cheminant J. R. Deering-Rice C. E. Massa C. B. Adhikari U. Noll J. Reilly C. A. (2025). Parenchymal and inflammatory responses to ozone exposure in the aging healthy and surfactant protein C mutant lung. Am. J. Physiol. Lung Cell Mol. Physiol. 328, L334–L349. 10.1152/ajplung.00261.2024 39832482 PMC12233909

[B13] Cheng C. Chen W. Jin H. Chen X. (2023). A review of single-cell RNA-Seq annotation, integration, and cell-cell communication. Cells 12, 1970. 10.3390/cells12151970 PMC10417635 37566049

[B14] Choudhary S. Satija R. (2022). Comparison and evaluation of statistical error models for scRNA-seq. Genome Biol. 23, 27. 10.1186/s13059-021-02584-9 35042561 PMC8764781

[B15] Clarke Z. A. Andrews T. S. Atif J. Pouyabahar D. Innes B. T. MacParland S. A. (2021). Tutorial: guidelines for annotating single-cell transcriptomic maps using automated and manual methods. Nat. Protoc. 16, 2749–2764. 10.1038/s41596-021-00534-0 34031612

[B16] Desta G. M. Birhanu A. G. (2024). Single-cell RNA sequencing: current progresses and future perspectives. Open Biotechnol. J. 18, e18740707311249. 10.2174/0118740707311249240604053619

[B17] Dimitrov D. Türei D. Garrido-Rodriguez M. Burmedi P. L. Nagai J. S. Boys C. (2022). Comparison of methods and resources for cell-cell communication inference from single-cell RNA-seq data. Nat. Commun. 13, 3224. 10.1038/s41467-022-30755-0 35680885 PMC9184522

[B18] Dimitrov D. Schäfer P. S. L. Farr E. Rodriguez-Mier P. Lobentanzer S. Badia-I-Mompel P. (2024). LIANA+ provides an all-in-one framework for cell-cell communication inference. Nat. Cell Biol. 26, 1613–1622. 10.1038/s41556-024-01469-w 39223377 PMC11392821

[B19] Duong T. E. Wu Y. Sos B. C. Dong W. Limaye S. Rivier L. H. (2022). A single-cell regulatory map of postnatal lung alveologenesis in humans and mice. Cell Genomics 2, 100108. 10.1016/j.xgen.2022.100108 PMC9012447 35434692

[B20] Efremova M. Vento-Tormo M. Teichmann S. A. Vento-Tormo R. (2020). CellPhoneDB: inferring cell-cell communication from combined expression of multi-subunit ligand-receptor complexes. Nat. Protoc. 15, 1484–1506. 10.1038/s41596-020-0292-x 32103204

[B21] Fujino N. Yamada M. Saito T. Konno S. Sugiura H. (2024). Human lung cell separation strategies for translational research. Am. J. Respir. Cell Mol. Biol. 71, 621–622. 10.1165/rcmb.2024-0338LE 39226578 PMC11568478

[B22] Germain P.-L. Sonrel A. Robinson M. D. (2020). pipeComp, a general framework for the evaluation of computational pipelines, reveals performant single cell RNA-seq preprocessing tools. Genome Biol. 21, 227. 10.1186/s13059-020-02136-7 32873325 PMC7465801

[B23] Ghaedi M. Shen Z. Y. Orangi M. Martinez-Gonzalez I. Wei L. Lu X. (2020). Single-cell analysis of RORα tracer mouse lung reveals ILC progenitors and effector ILC2 subsets. J. Exp. Med. 217 (jem), 20182293. 10.1084/jem.20182293 31816636 PMC7062532

[B24] Guo M. Morley M. P. Jiang C. Wu Y. Li G. Du Y. (2023). Guided construction of single cell reference for human and mouse lung. Nat. Commun. 14, 4566. 10.1038/s41467-023-40173-5 37516747 PMC10387117

[B25] Habermann A. C. Gutierrez A. J. Bui L. T. Yahn S. L. Winters N. I. Calvi C. L. (2020). Single-cell RNA sequencing reveals profibrotic roles of distinct epithelial and mesenchymal lineages in pulmonary fibrosis. Sci. Adv. 6, eaba1972. 10.1126/sciadv.aba1972 32832598 PMC7439444

[B26] Hafemeister C. Satija R. (2019). Normalization and variance stabilization of single-cell RNA-seq data using regularized negative binomial regression. Genome Biol. 20, 296. 10.1186/s13059-019-1874-1 31870423 PMC6927181

[B27] Haghverdi L. Lun A. T. L. Morgan M. D. Marioni J. C. (2018). Batch effects in single-cell RNA-sequencing data are corrected by matching mutual nearest neighbors. Nat. Biotechnol. 36, 421–427. 10.1038/nbt.4091 29608177 PMC6152897

[B28] Hao Y. Hao S. Andersen-Nissen E. Mauck W. M. Zheng S. Butler A. (2021). Integrated analysis of multimodal single-cell data. Cell 184, 3573–3587.e29. 10.1016/j.cell.2021.04.048 34062119 PMC8238499

[B29] Hao Y. Stuart T. Kowalski M. H. Choudhary S. Hoffman P. Hartman A. (2024). Dictionary learning for integrative, multimodal and scalable single-cell analysis. Nat. Biotechnol. 42, 293–304. 10.1038/s41587-023-01767-y 37231261 PMC10928517

[B30] Hou W. Ji Z. Ji H. Hicks S. C. (2020). A systematic evaluation of single-cell RNA-sequencing imputation methods. Genome Biol. 21, 218. 10.1186/s13059-020-02132-x 32854757 PMC7450705

[B31] Jin S. Guerrero-Juarez C. F. Zhang L. Chang I. Ramos R. Kuan C.-H. (2021). Inference and analysis of cell-cell communication using CellChat. Nat. Commun. 12, 1088. 10.1038/s41467-021-21246-9 33597522 PMC7889871

[B32] Jin S. Plikus M. V. Nie Q. (2025). CellChat for systematic analysis of cell-cell communication from single-cell transcriptomics. Nat. Protoc. 20, 180–219. 10.1038/s41596-024-01045-4 39289562

[B33] Jirmo A. C. Grychtol R. Gaedcke S. Liu B. DeStefano S. Happle C. (2023). Single cell RNA sequencing reveals distinct clusters of Irf8-expressing pulmonary conventional dendritic cells. Front. Immunol. 14, 1127485. 10.3389/fimmu.2023.1127485 37251386 PMC10213693

[B34] Joshi N. Watanabe S. Verma R. Jablonski R. P. Chen C.-I. Cheresh P. (2020). A spatially restricted fibrotic niche in pulmonary fibrosis is sustained by M-CSF/M-CSFR signalling in monocyte-derived alveolar macrophages. Eur. Respir. J. 55, 1900646. 10.1183/13993003.00646-2019 31601718 PMC6962769

[B35] Korsunsky I. Millard N. Fan J. Slowikowski K. Zhang F. Wei K. (2019). Fast, sensitive and accurate integration of single-cell data with Harmony. Nat. Methods 16, 1289–1296. 10.1038/s41592-019-0619-0 31740819 PMC6884693

[B36] Lopez R. Regier J. Cole M. B. Jordan M. I. Yosef N. (2018). Deep generative modeling for single-cell transcriptomics. Nat. Methods 15, 1053–1058. 10.1038/s41592-018-0229-2 30504886 PMC6289068

[B37] Luecken M. D. Theis F. J. (2019). Current best practices in single-cell RNA-seq analysis: a tutorial. Mol. Syst. Biol. 15, e8746. 10.15252/msb.20188746 31217225 PMC6582955

[B38] Luecken M. D. Büttner M. Chaichoompu K. Danese A. Interlandi M. Mueller M. F. (2022). Benchmarking atlas-level data integration in single-cell genomics. Nat. Methods 19, 41–50. 10.1038/s41592-021-01336-8 34949812 PMC8748196

[B39] McGinnis C. S. Murrow L. M. Gartner Z. J. (2019). DoubletFinder: doublet detection in single-cell RNA sequencing data using artificial nearest neighbors. Cell Syst. 8, 329–337.e4. 10.1016/j.cels.2019.03.003 30954475 PMC6853612

[B40] McInnes L. Healy J. Melville J. (2020). UMAP: Uniform Manifold Approximation and Projection for Dimension Reduction. 10.48550/arXiv.1802.03426

[B41] Molla Desta G. Birhanu A. G. (2025). Advancements in single-cell RNA sequencing and spatial transcriptomics: transforming biomedical research. Acta Biochim. Pol. 72, 13922. 10.3389/abp.2025.13922 39980637 PMC11835515

[B42] Moos P. J. Cheminant J. R. Cowman S. Noll J. Wang Q. Musci T. (2024). Spatial and phenotypic heterogeneity of resident and monocyte-derived macrophages during inflammatory exacerbations leading to pulmonary fibrosis. Front. Immunol. 15, 1425466. 10.3389/fimmu.2024.1425466 39100672 PMC11294112

[B43] Moos P. Cheminant J. Adhikari U. Venosa A. (2024). Transcriptomic-based roadmap to the healthy and ozone-exposed lung. Curr. Opin. Toxicol. 37, 100445. 10.1016/j.cotox.2023.100445 38187954 PMC10769160

[B44] Negretti N. M. Plosa E. J. Benjamin J. T. Schuler B. A. Habermann A. C. Jetter C. S. (2021). A single-cell atlas of mouse lung development. Development 148, dev199512. 10.1242/dev.199512 34927678 PMC8722390

[B45] Nureki S.-I. Tomer Y. Venosa A. Katzen J. Russo S. J. Jamil S. (2018). Expression of mutant Sftpc in murine alveolar epithelia drives spontaneous lung fibrosis. J. Clin. Invest. 128, 4008–4024. 10.1172/JCI99287 29920187 PMC6118576

[B46] Quintanal-Villalonga Á. Chan J. M. Masilionis I. Gao V. R. Xie Y. Allaj V. (2022). Protocol to dissociate, process, and analyze the human lung tissue using single-cell RNA-seq. Star. Protoc. 3, 101776. 10.1016/j.xpro.2022.101776 36313536 PMC9597186

[B47] Reyfman P. A. Walter J. M. Joshi N. Anekalla K. R. McQuattie-Pimentel A. C. Chiu S. (2019). Single-cell transcriptomic analysis of human lung provides insights into the pathobiology of pulmonary fibrosis. Am. J. Respir. Crit. Care Med. 199, 1517–1536. 10.1164/rccm.201712-2410OC 30554520 PMC6580683

[B48] Rodriguez L. R. Tang S. Y. Roque Barboza W. Murthy A. Tomer Y. Cai T.-Q. (2023). PGF2α signaling drives fibrotic remodeling and fibroblast population dynamics in mice. JCI Insight 8, e172977. 10.1172/jci.insight.172977 PMC10807712 37934604

[B49] Rodríguez L. R. Alysandratos K.-D. Katzen J. Murthy A. Roque Barboza W. Tomer Y. (2025). Impaired AMPK control of alveolar epithelial cell metabolism promotes pulmonary fibrosis. JCI Insight 10, e182578. 10.1172/jci.insight.182578 40591409 PMC12333953

[B50] Sikkema L. Ramírez-Suástegui C. Strobl D. C. Gillett T. E. Zappia L. Madissoon E. (2023). An integrated cell atlas of the lung in health and disease. Nat. Med. 29, 1563–1577. 10.1038/s41591-023-02327-2 37291214 PMC10287567

[B51] Song Y. Miao Z. Brazma A. Papatheodorou I. (2023). Benchmarking strategies for cross-species integration of single-cell RNA sequencing data. Nat. Commun. 14, 6495. 10.1038/s41467-023-41855-w 37838716 PMC10576752

[B52] Tabula Muris Consortium (2018). Overall coordination, Logistical coordination, Organ collection and processing, Library preparation and sequencing, Computational data analysis, et al. Single-cell transcriptomics of 20 mouse organs creates a Tabula Muris. Nature 562, 367–372. 10.1038/s41586-018-0590-4 30283141 PMC6642641

[B53] Tabula Muris Consortium (2020). A single-cell transcriptomic atlas characterizes ageing tissues in the mouse. Nature 583, 590–595. 10.1038/s41586-020-2496-1 32669714 PMC8240505

[B54] Tirosh I. Izar B. Prakadan S. M. Wadsworth M. H. Treacy D. Trombetta J. J. (2016). Dissecting the multicellular ecosystem of metastatic melanoma by single-cell RNA-seq. Science 352, 189–196. 10.1126/science.aad0501 27124452 PMC4944528

[B55] Townes F. W. Hicks S. C. Aryee M. J. Irizarry R. A. (2019). Feature selection and dimension reduction for single-cell RNA-seq based on a multinomial model. Genome Biol. 20, 295. 10.1186/s13059-019-1861-6 31870412 PMC6927135

[B56] Tran H. T. N. Ang K. S. Chevrier M. Zhang X. Lee N. Y. S. Goh M. (2020). A benchmark of batch-effect correction methods for single-cell RNA sequencing data. Genome Biol. 21, 12. 10.1186/s13059-019-1850-9 31948481 PMC6964114

[B57] Troulé K. Petryszak R. Cakir B. Cranley J. Harasty A. Prete M. (2025). CellPhoneDB v5: inferring cell-cell communication from single-cell multiomics data. Nat. Protoc. 20, 3412–3440. 10.1038/s41596-024-01137-1 40133495

[B58] Tsukui T. Sun K.-H. Wetter J. B. Wilson-Kanamori J. R. Hazelwood L. A. Henderson N. C. (2020). Collagen-producing lung cell atlas identifies multiple subsets with distinct localization and relevance to fibrosis. Nat. Commun. 11, 1920. 10.1038/s41467-020-15647-5 32317643 PMC7174390

[B59] Tung P.-Y. Blischak J. D. Hsiao C. J. Knowles D. A. Burnett J. E. Pritchard J. K. (2017). Batch effects and the effective design of single-cell gene expression studies. Sci. Rep. 7, 39921. 10.1038/srep39921 28045081 PMC5206706

[B60] Waltman L. Van Eck N. J. (2013). A smart local moving algorithm for large-scale modularity-based community detection. Eur. Phys. J. B 86, 471. 10.1140/epjb/e2013-40829-0

[B61] Wang Y. Wang R. Zhang S. Song S. Jiang C. Han G. (2019). iTALK: An R Package to Characterize and Illustrate Intercellular Communication. 10.1101/507871

[B62] Wang S. Li J. Wu C. Lei Z. Wang T. Huang X. (2024). Single-Cell RNA sequencing reveals monocyte-derived interstitial macrophages with a pro-fibrotic phenotype in bleomycin-induced pulmonary fibrosis. Int. J. Mol. Sci. 25, 11669. 10.3390/ijms252111669 39519222 PMC11545836

[B63] Wei W. Xia X. Li T. Chen Q. Feng X. (2024). Shaoxia: a web-based interactive analysis platform for single cell RNA sequencing data. BMC Genomics 25, 402. 10.1186/s12864-024-10322-1 38658838 PMC11040744

[B64] Zhang L. Gao S. White Z. Dai Y. Malik A. B. Rehman J. (2022). Single-cell transcriptomic profiling of lung endothelial cells identifies dynamic inflammatory and regenerative subpopulations. JCI Insight 7, e158079. 10.1172/jci.insight.158079 35511435 PMC9220950

[B65] Zhao R. Lu J. Li Y. Zhou W. Zhao N. Ji H. (2025). A systematic evaluation of highly variable gene selection methods for single-cell RNA-sequencing. Genome Biol. 26, 424. 10.1186/s13059-025-03887-x 41382205 PMC12699822

[B66] Zilionis R. Engblom C. Pfirschke C. Savova V. Zemmour D. Saatcioglu H. D. (2019). Single-cell transcriptomics of human and mouse lung cancers reveals conserved myeloid populations across individuals and species. Immunity 50, 1317–1334.e10. 10.1016/j.immuni.2019.03.009 30979687 PMC6620049

