# Supplementary material for: A single-cell atlas and Shiny-based framework for murine lung injury and remodeling
Source: Front Bioinform. 2026 Sep 21;6:1886929. doi: 10.3389/fbinf.2026.1886929 (PMC13635373; doi:10.3389/fbinf.2026.1886929)
Supplement: Supplementary file 1 [file DataSheet1.pdf]

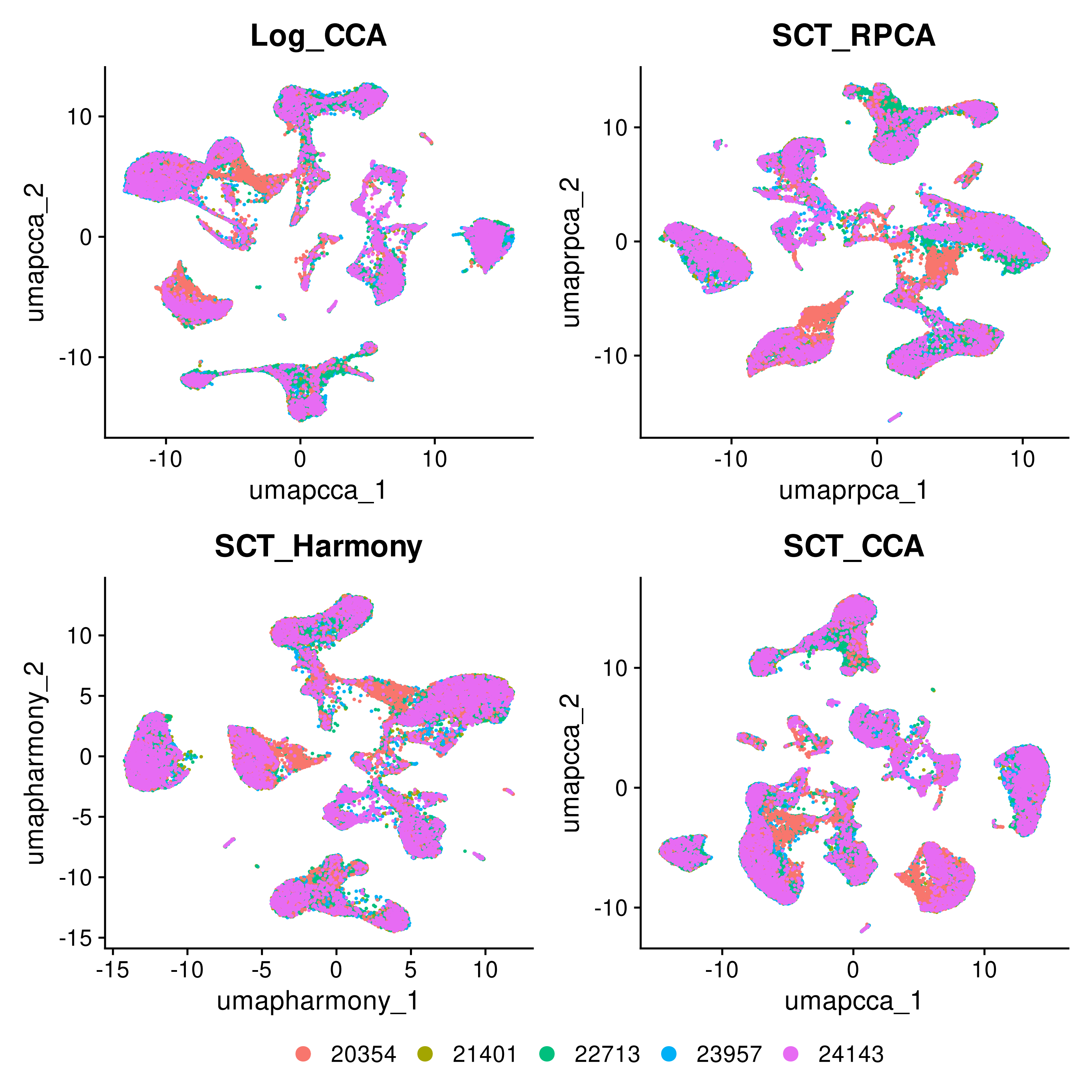

Log_CCA
umapcca_2
umapcca_1
-10
0
10
SCT_RPCA
umaprpca_2
umaprpca_1
SCT_Harmony
umapharmony_2
umapharmony_1
-15
-10
-5
0
5
10
SCT_CCA
umapcca_2
umapcca_1
20354
21401
22713
23957
24143
