# Supplementary material for: A single-cell atlas and Shiny-based framework for murine lung injury and remodeling
Source: Front Bioinform. 2026 Sep 21;6:1886929. doi: 10.3389/fbinf.2026.1886929 (PMC13635373; doi:10.3389/fbinf.2026.1886929)
Supplement: Supplementary file 4 [file DataSheet4.pdf]

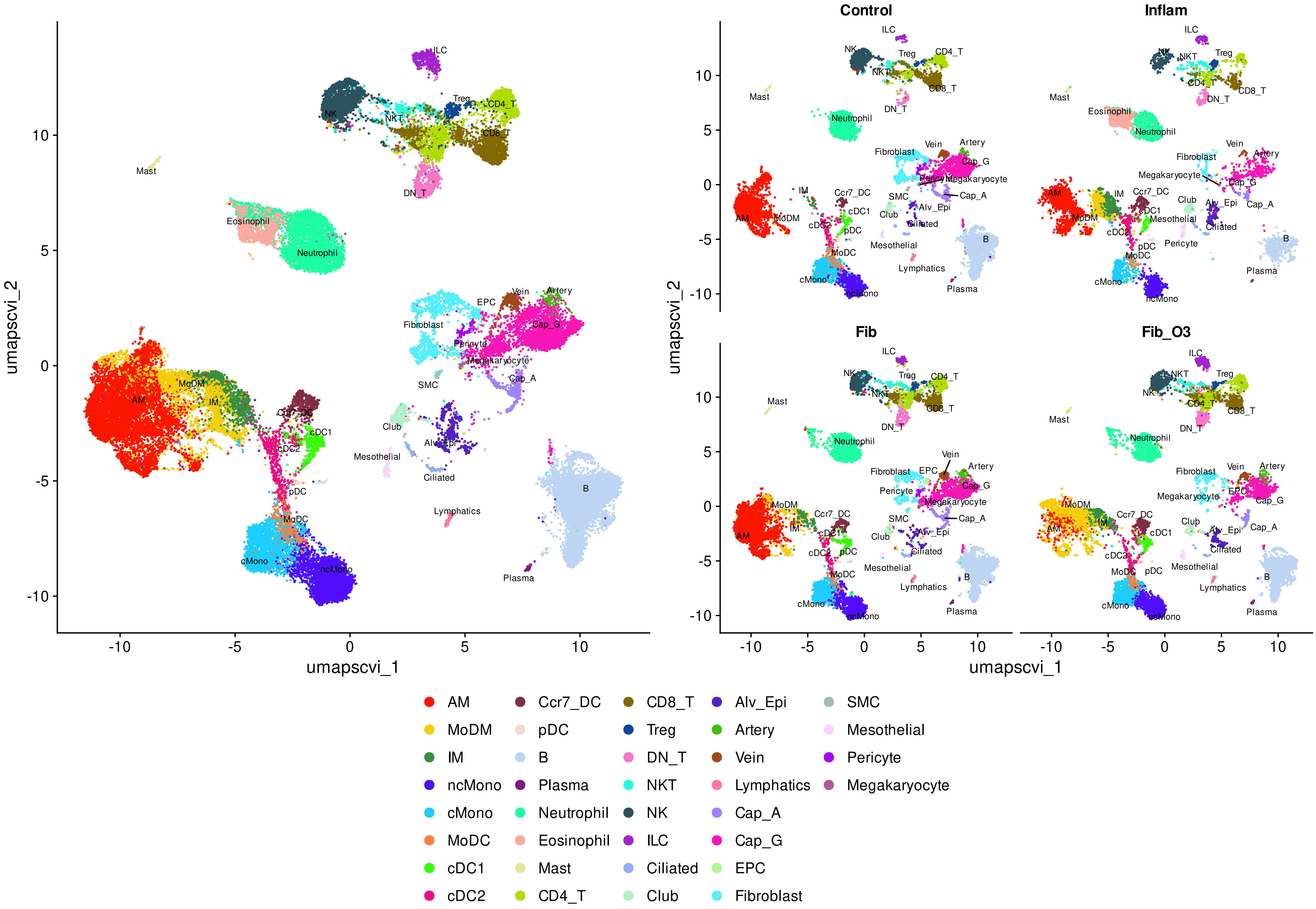

Control
Inflam
Fib
Fib_O3
umapscvi_1
umapscvi_2
AM
MoDM
IM
ncMono
cMono
MoDC
cDC1
cDC2
Ccr7_DC
pDC
B
Plasma
Neutrophil
Eosinophil
Mast
CD4_T
CD8_T
Treg
DN_T
NKT
NK
ILC
Ciliated
Club
Alv_Epi
Artery
Vein
Lymphatics
Cap_A
Cap_G
EPC
Fibroblast
SMC
Mesothelial
Pericyte
Megakaryocyte
