# Supplementary material for: A single-cell atlas and Shiny-based framework for murine lung injury and remodeling
Source: Front Bioinform. 2026 Sep 21;6:1886929. doi: 10.3389/fbinf.2026.1886929 (PMC13635373; doi:10.3389/fbinf.2026.1886929)
Supplement: Supplementary file 5 [file DataSheet5.pdf]

A

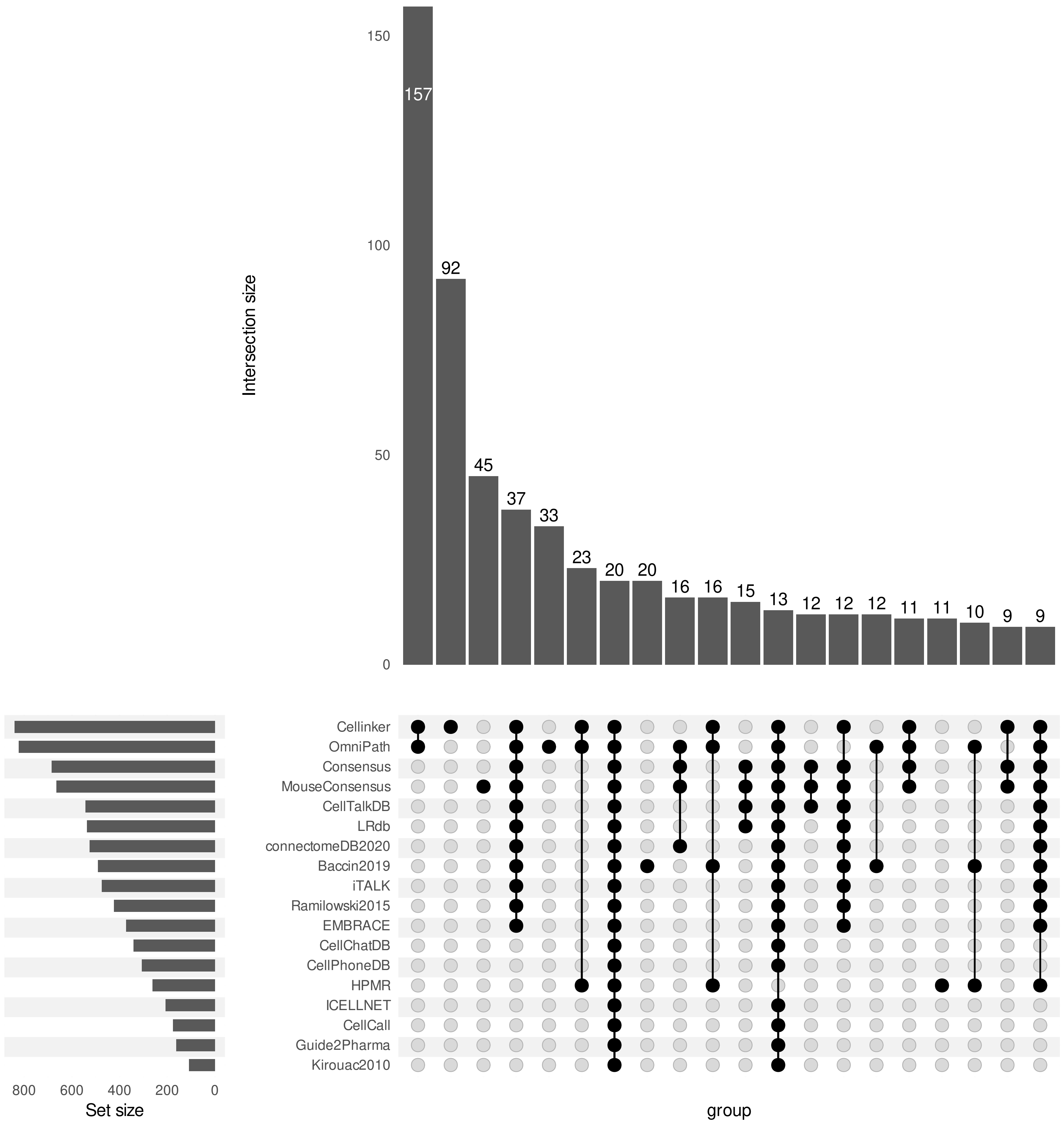

157
92
45
37
33
23
20
20
16
16
15
13
12
12
12
11
11
10
9
9
Intersection size
150
100
50
0
Cellinker
OmniPath
Consensus
MouseConsensus
CellTalkDB
LRdb
connectomeDB2020
Baccin2019
iTALK
Ramilowski2015
EMBRACE
CellChatDB
CellPhoneDB
HPMR
ICELLNET
CellCall
Guide2Pharma
Kirouac2010
800
600
400
200
0
Set size
group

B

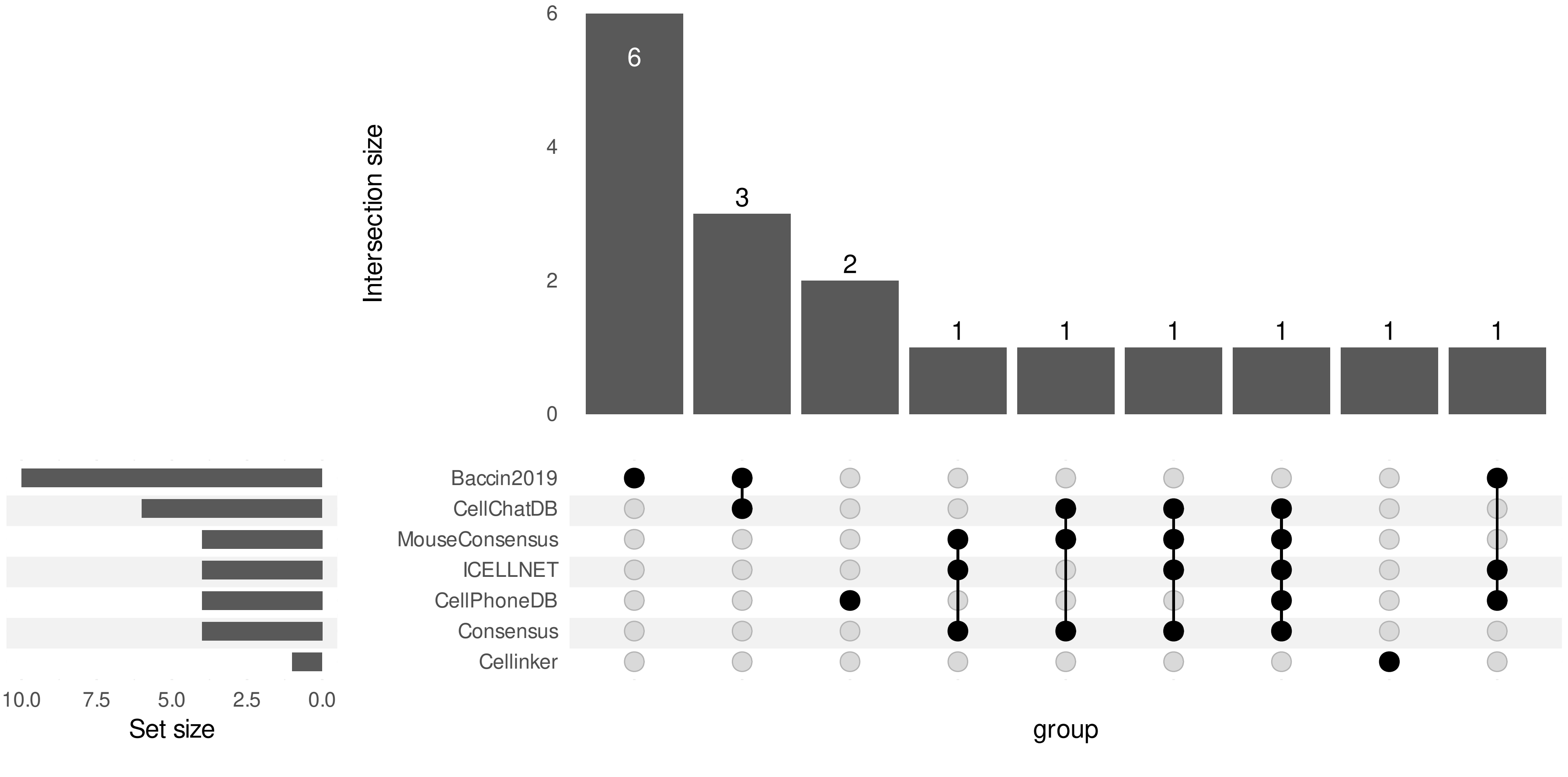

6
3
2
1
1
1
1
1
1
Intersection size
6
4
2
0
Baccin2019
CellChatDB
MouseConsensus
ICELLNET
CellPhoneDB
Consensus
Cellinker
10.0
7.5
5.0
2.5
0.0
Set size
group
