# Supplementary material for: A single-cell atlas and Shiny-based framework for murine lung injury and remodeling
Source: Front Bioinform. 2026 Sep 21;6:1886929. doi: 10.3389/fbinf.2026.1886929 (PMC13635373; doi:10.3389/fbinf.2026.1886929)
Supplement: Supplementary file 6 [file DataSheet6.pdf]

A

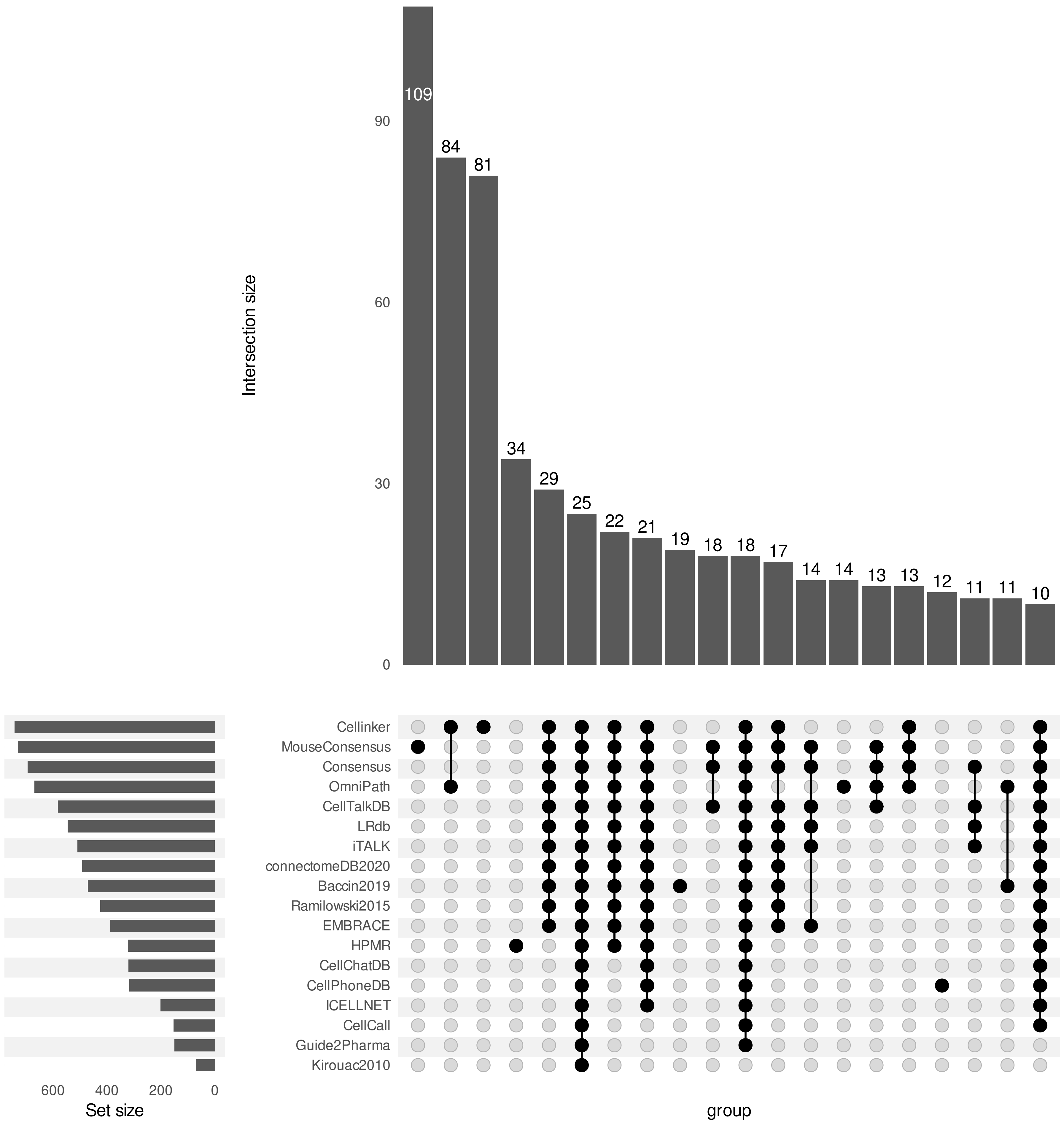
Intersection size
109
84
81
34
29
25
22
21
19
18
18
17
14
14
13
13
12
11
11
10
0
30
60
90
Cellinker
MouseConsensus
Consensus
OmniPath
CellTalkDB
LRdb
iTALK
connectomeDB2020
Baccin2019
Ramilowski2015
EMBRACE
HPMR
CellChatDB
CellPhoneDB
ICELLNET
CellCall
Guide2Pharma
Kirouac2010
600
400
200
0
Set size
group

B

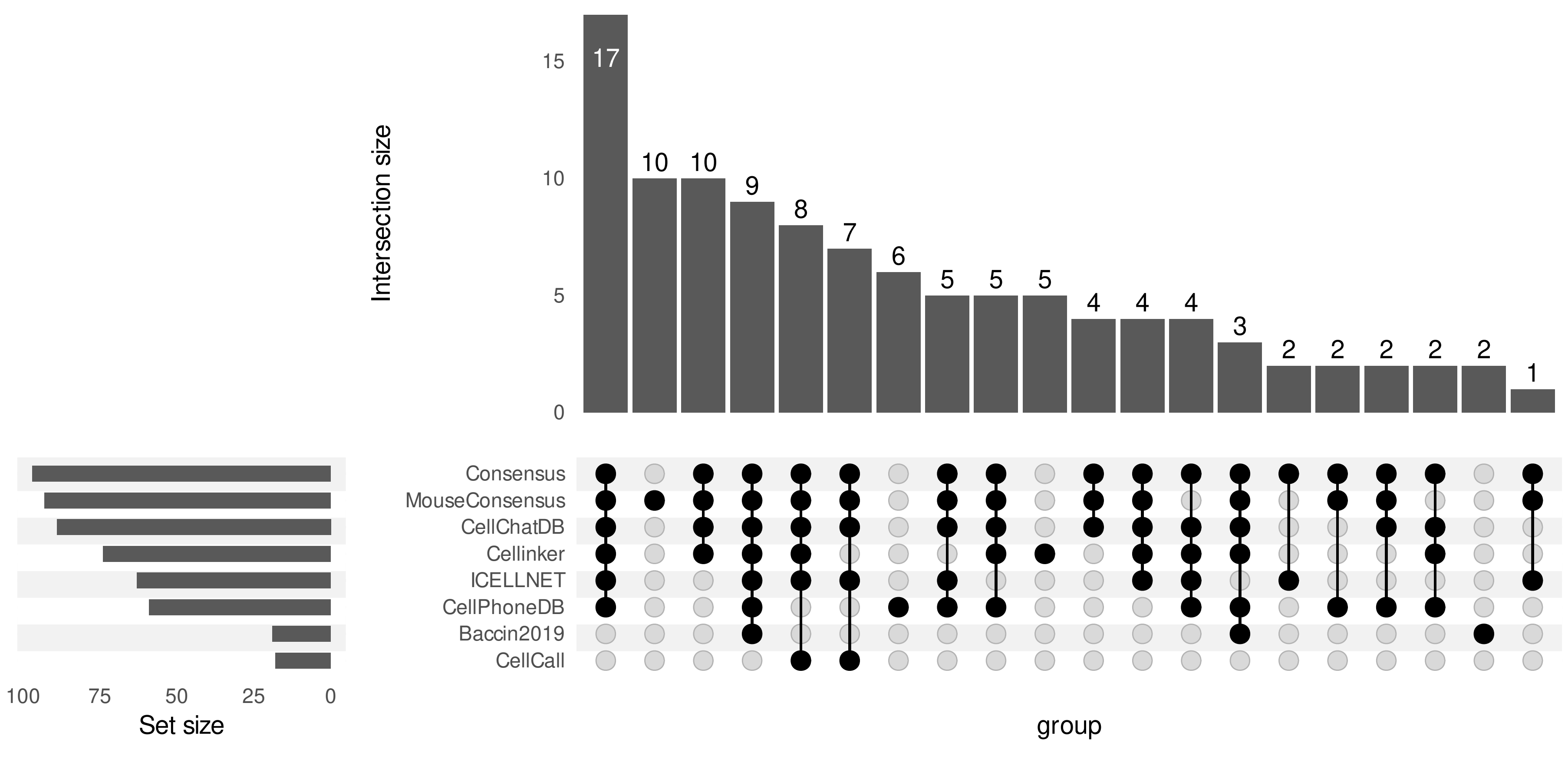
Intersection size
17
10
10
9
8
7
6
5
5
5
4
4
4
3
2
2
2
2
2
1
0
5
10
15
Consensus
MouseConsensus
CellChatDB
Cellinker
ICELLNET
CellPhoneDB
Baccin2019
CellCall
100
75
50
25
0
Set size
group
